# Symbiont-Mediated Defense against Legionella pneumophila in Amoebae

**DOI:** 10.1128/mBio.00333-19

**Published:** 2019-05-14

**Authors:** Lena König, Cecilia Wentrup, Frederik Schulz, Florian Wascher, Sarah Escola, Michele S. Swanson, Carmen Buchrieser, Matthias Horn

**Affiliations:** a Centre for Microbiology and Environmental Systems Science, University of Vienna, Vienna, Austria; b Biologie des Bactéries Intracellulaires, Institut Pasteur, Paris, France; c CNRS, UMR 3525, Paris, France; d Department of Microbiology and Immunology, University of Michigan, Ann Arbor, Michigan, USA; University of Zürich; University of Hawaii at Manoa

**Keywords:** amoeba, *Legionella*, antimicrobial defense, coinfection, endosymbionts, protists

## Abstract

Legionella pneumophila is an important opportunistic pathogen for which environmental reservoirs are crucial for the infection of humans. In the environment, free-living amoebae represent key hosts providing nutrients and shelter for highly efficient intracellular proliferation of L. pneumophila, which eventually leads to lysis of the protist. However, the significance of other bacterial players for L. pneumophila ecology is poorly understood. In this study, we used a ubiquitous amoeba and bacterial endosymbiont to investigate the impact of this common association on L. pneumophila infection. We demonstrate that L. pneumophila proliferation was severely suppressed in Acanthamoeba castellanii harboring the chlamydial symbiont Protochlamydia amoebophila. The amoebae survived the infection and were able to resume growth. Different environmental amoeba isolates containing the symbiont were equally well protected as different L. pneumophila isolates were diminished, suggesting ecological relevance of this symbiont-mediated defense. Furthermore, protection was not mediated by impaired L. pneumophila uptake. Instead, we observed reduced virulence of L. pneumophila released from symbiont-containing amoebae. Pronounced gene expression changes in the presence of the symbiont indicate that interference with the transition to the transmissive phase impedes the L. pneumophila infection. Finally, our data show that the defensive response of amoebae harboring P. amoebophila leaves the amoebae with superior fitness reminiscent of immunological memory. Given that mutualistic associations between bacteria and amoebae are widely distributed, P. amoebophila and potentially other amoeba endosymbionts could be key in shaping environmental survival, abundance, and virulence of this important pathogen, thereby affecting the frequency of human infection.

## Abstract (precis)

Bacterial pathogens are generally investigated in the context of disease. To prevent outbreaks, it is essential to understand their lifestyle and interactions with other microbes in their natural environment. Legionella pneumophila is an important human respiratory pathogen that survives and multiplies in biofilms or intracellularly within protists, such as amoebae. Importantly, transmission to humans occurs from these environmental sources. *Legionella* infection generally leads to rapid host cell lysis. It was therefore surprising to observe that amoebae, including fresh environmental isolates, were well protected during *Legionella* infection when the bacterial symbiont Protochlamydia amoebophila was also present. *Legionella* was not prevented from invading amoebae but was impeded in its ability to develop fully virulent progeny and were ultimately cleared in the presence of the symbiont. This study highlights how ecology and virulence of an important human pathogen is affected by a defensive amoeba symbiont, with possibly major consequences for public health.

## INTRODUCTION

Free-living amoebae like *Acanthamoeba* are ubiquitous in soil and water environments, in which they prey on bacteria, thereby controlling bacterial populations and enhancing nutrient recycling (1, 2). Importantly, they are commonly found in anthropogenic water systems such as drinking and industrial water, where they graze on biofilms and interact with a diverse microbial community (3–6). Apart from bacteria as a food source, free-living amoebae are commonly associated with facultative or obligate intracellular bacteria that survive phagocytosis. These microbes either transiently infect amoebae, exploit their host for multiplication, and finally lyse them (acting as amoeba pathogens), or they establish long-term stable associations as they are strictly dependent on amoebae as hosts for intracellular replication (amoeba endosymbionts) (7–11). When conditions turn unfavorable, acanthamoebae differentiate from the vegetative trophozoite stage to a highly resistant cyst form (2). Several bacterial pathogens and endosymbionts have been reported to survive encystment, facilitating dispersal and protection from adverse conditions (7, 12–18).

Amoeba pathogens are frequently also human pathogens (10), the prime example being the facultative intracellular bacterium Legionella pneumophila, an important cause of community- and hospital-acquired pneumonia termed Legionnaires’ disease (19). The intracellular life cycle of L. pneumophila is strikingly similar between amoebae and mammalian macrophages: host cell-specific attachment is followed by uptake via “coiling phagocytosis” and a partly conserved activation of signaling pathways. They evade the endocytic pathway, delay vacuole acidification, remodel the phagosome to a *Legionella*-containing vacuole (LCV), and modulate host cellular processes, thereby allowing efficient intravacuolar replication. During late stages of infection, L. pneumophila transitions into the virulent, transmissive stage, escapes into the host cytosol, and exits the host cell by lysis (20–23).

In the environment, L. pneumophila is thought to most efficiently replicate within free-living amoebae, leading to the release of highly virulent bacteria primed for the infection of humans (21, 24). Consistent with this, L. pneumophila was found to cooccur with free-living amoebae in various aquatic environments (5, 10, 25, 26). Within cysts, amoebae also grant protection from harsh environmental conditions, and they facilitate resuscitation of viable but nonculturable L. pneumophila (12, 21, 27). Because the main route of transmission of L. pneumophila to humans is from the environment, outbreaks might be controlled by targeting free-living amoebae instead of L. pneumophila directly (28, 29).

Bacterial endosymbionts of acanthamoebae are diverse and widespread, and in particular, endosymbionts related to the human pathogen Chlamydia trachomatis are frequently found in *Acanthamoeba* isolates (7, 10, 13, 25, 30–36). Among these environmental chlamydiae, Protochlamydia amoebophila has been studied to some extent (37–41). Originally detected as symbionts in an *Acanthamoeba* isolate from soil (7, 13), these bacteria were shown to thrive within a range of different *Acanthamoeba* strains (42). Like the human pathogens, P. amoebophila follows a characteristic developmental cycle (30, 41), and this obligate intracellular lifestyle is believed to be several hundred million years old (43). Other *Acanthamoeba* endosymbionts closely related to P. amoebophila have been found (25, 33, 35, 36, 44), and rRNA gene sequences assigned to the same chlamydial family (*Parachlamydiaceae*) have been detected in diverse environments (45), suggesting that, like *Acanthamoeba* hosts, *Protochlamydia* symbionts are ubiquitous.

Despite sharing the same host, the interaction between amoeba pathogens and symbionts has rarely been investigated. In particular, the impact of bacterial symbionts on the environmental niche of L. pneumophila is largely unclear. Recent findings, however, indicate that amoebae harboring a *Neochlamydia* species endosymbiont are more resistant to infection with L. pneumophila (46, 47). Here, we explored the effect of P. amoebophila endosymbionts on various amoeba hosts in the face of L. pneumophila infection. Our results demonstrate that (long-term) laboratory-maintained as well as freshly isolated environmental *Acanthamoeba* strains survive infection either with laboratory or environmental L. pneumophila strains in the presence of P. amoebophila. We provide evidence that this symbiont-mediated defense is caused by interference with normal L. pneumophila development. Together, these findings identify bacterial endosymbionts of amoebae as an important factor in the ecology of L. pneumophila, with a fundamental impact on environmental survival and transmission of L. pneumophila to humans.

## RESULTS

### Amoeba survival of L. pneumophila infection in the presence of chlamydial symbionts.

To assess the impact of chlamydial endosymbionts on L. pneumophila infection of amoebae, we first established genetically identical (isogenic) A. castellanii Neff cultures with and without P. amoebophila as the symbiont. We next evaluated the effect of the symbiont on the growth rate of its host. P. amoebophila remains stably associated with its acanthamoeba host and does not cause lysis, yet the symbiont slows down amoeba growth irrespective of the incubation temperature (see Fig. S1A in the supplemental material). Thus, harboring the symbiont *per se* does not increase amoeba fitness in terms of reproductive success, but P. amoebophila spreads efficiently through uninfected amoeba populations (Fig. S1B).

10.1128/mBio.00333-19.3 FIG S1 Amoeba growth and spread of P. amoebophila infection. (A) A. castellanii Neff amoebae with and without the endosymbiont P. amoebophila (*Pam*) were seeded at low densities to avoid contact-dependent inhibition of growth (Text S1), and growth was monitored over 4 days at two different temperatures. Data points from all three replicates are plotted (filled circles), and exponential functions were applied to model growth (lines). Mean doubling times (*t_d_* [h]) under each condition are depicted on the right. Independent of the temperature, symbiont-free amoebae replicated faster than amoebae with the symbiont, indicating that harboring the symbiont impairs host fitness. Furthermore, as symbiont-harboring amoebae replicated continuously without notable lysis over the time examined, the symbiont is transmitted vertically, and the association is stable. (B) Amoebae with and without the symbiont were mixed (1:10), and the proportion of P. amoebophila-containing amoebae was monitored over three days (30°C) (Text S1). Data points from all three replicates are plotted (filled circles), and logistic curves (lines) best represent the course of increasing proportion of symbiont-containing amoebae and decreasing proportion of uninfected amoebae. All or nearly all amoebae harbored symbionts after 69 h of cocultivation in all replicates, demonstrating horizontal (in addition to vertical) transmission of P. amoebophila. Download FIG S1, PDF file, 0.05 MB. Copyright © 2019 König et al. 2019 König et al. This content is distributed under the terms of the Creative Commons Attribution 4.0 International license.

10.1128/mBio.00333-19.10 TEXT S1 Supplemental materials and methods. Download Text S1, DOCX file, 0.01 MB. Copyright © 2019 König et al. 2019 König et al. This content is distributed under the terms of the Creative Commons Attribution 4.0 International license.

**FIG 1:**
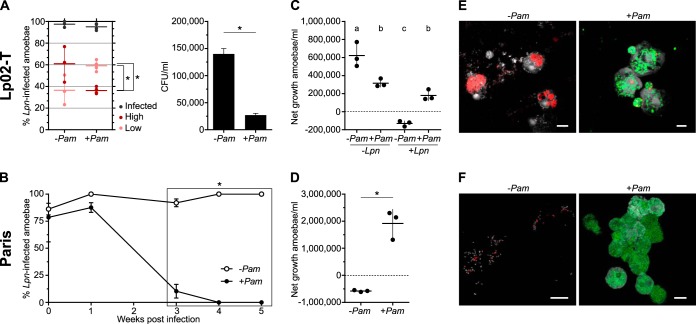
Reduced L. pneumophila infection and increased survival of Acanthamoeba castellanii Neff in the presence of P. amoebophila symbionts. (A) L. pneumophila Lp02-T load in the absence (−) and presence (+) of P. amoebophila (*Pam*) at 5 weeks postinfection (wpi) was measured as the proportion of amoebae containing L. pneumophila FISH signals (infected), the proportion of amoebae containing >5 L. pneumophila signals (high), and the proportion of amoebae containing only 1 to 5 L. pneumophila signals (low). In addition, growth of Lp02-T was assessed by determining CFU per ml at 5 wpi. The difference between −*Pam* and +*Pam* was statistically significant in comparisons of low and high infection levels (*, *P* < 0.05 by unpaired *t* test) and CFU/ml (*, *P* < 0.001 by unpaired *t* test). (B) L. pneumophila Paris infection levels were also determined via FISH, but here the course of infection over 5 weeks is shown because the endpoints were 0% and 100% infected amoebae. Two-way analysis of variance (ANOVA) combined with Tukey’s test was applied to compare the two curves, and significantly different time points are indicated (box marked by an asterisk, *P* < 0.001). (C and D) Amoeba growth at 5 weeks after L. pneumophila infection is expressed as the difference between start and endpoint amoeba numbers. In the case of strain Lp02-T (C), symbiont-free and symbiont-containing amoebae not infected with L. pneumophila (−*Lpn*) served as additional controls. Lowercase letters denote significantly distinct statistical groups (*P* < 0.01 by one-way ANOVA and Tukey’s test). (D) Statistical testing in the case of strain Paris was done using the unpaired *t* test (*, *P* < 0.01). In panels A, C, and D, horizontal lines denote means and error bars show standard deviations from three biological replicates. (E and F) The infection status of L. pneumophila Lp02-T (E) and Paris (F) at 5 wpi was visualized by FISH (red, LEGPNE1 probe specific for L. pneumophila; green, Chls-523 probe specific for chlamydiae) and DAPI staining (gray). Infection experiments were carried out at 30°C for L. pneumophila Lp02-T (MOI of 20) and at 20°C for L. pneumophila Paris (MOI of 0.5). Scale bars, 10 μm.

We next challenged A. castellanii Neff with and without symbionts with two different Legionella pneumophila strains (L. pneumophila Paris and Lp02-T), both of which originate from outbreaks of Legionnaires’ disease (48, 49). Most notably, irrespective of the L. pneumophila strain, multiplicity of infection (MOI), incubation time, and temperature, harboring the symbiont always proved to result in a decreased L. pneumophila load compared to that of the symbiont-free control (Table S1). The impact of the symbiont is demonstrated by both a significantly lower proportion of (highly) infected amoebae as well as lower L. pneumophila cell numbers at either 1 or 5 weeks postinfection (wpi) observed in ten different experimental setups (Fig. 1A and B and Table S1 and Fig. S2A), even though L. pneumophila was able to replicate at the beginning of the experiment when symbionts were present (Fig. S2B and S3). Of note, L. pneumophila was observed within amoeba cells (trophozoites) as early as 2 h postinfection (hpi), and L. pneumophila-containing vacuoles and P. amoebophila inclusions remained well separated during coinfection (Fig. S3).

10.1128/mBio.00333-19.1 TABLE S1 Summary of infection experiments. Download Table S1, DOCX file, 0.02 MB. Copyright © 2019 König et al. 2019 König et al. This content is distributed under the terms of the Creative Commons Attribution 4.0 International license.

10.1128/mBio.00333-19.4 FIG S2 Decrease of L. pneumophila cell numbers in the presence of the symbiont. L. pneumophila numbers are shown as CFU/ml. (A) L. pneumophila numbers are drastically reduced already one week postinfection when A. castellanii Neff harbors symbionts. The difference is statistically significant for both L. pneumophila strains measured (*, *P* < 0.05, unpaired *t* test). (B) L. pneumophila Lp02-T within A. castellanii Neff initially grows intracellularly before numbers decrease when the endosymbiont is present. (A and B) Error bars show the standard deviations from three biological replicates. Infections of A. castellanii Neff with L. pneumophila Lp02-T were carried out at 30°C (MOI of 20); infections with L. pneumophila Paris were conducted at 20°C (MOI of 10). –*Pam*, P. amoebophila absent; +*Pam*, P. amoebophila present. Download FIG S2, PDF file, 0.05 MB. Copyright © 2019 König et al. 2019 König et al. This content is distributed under the terms of the Creative Commons Attribution 4.0 International license.

10.1128/mBio.00333-19.5 FIG S3 Course of L. pneumophila infection in the presence and absence of P. amoebophila monitored by FISH. (A) Amoebae harboring the symbiont do not block L. pneumophila infection at 2 hpi and still support L. pneumophila growth. A. castellanii Neff amoebae with (+Pam) and without (−*Pam*) the symbiont were infected with L. pneumophila Lp02-T (upper; MOI of 20, 30°C) or Paris (lower; MOI of 10, 20°C), and the course of infection was monitored targeting L. pneumophila (red/pink, LEGPNE1 probe) and P. amoebophila (green, Chls523 probe). Amoeba outlines (white lines) are based on corresponding phase contrast images (upper) or FISH staining of the amoebae (lower). Note that amoeba cell sizes differed at 48 and 96 hpi (unpaired *t* test, *P* < 0.05), with infected amoeba carrying the symbiont being smaller on average (13 to 17 μm versus 19 to 26 μm for strain Lp02-T and 10 to 11 μm versus 12 to 17 μm for strain Paris). Scale bars, 20 μm. (B) L. pneumophila and P. amoebophila always appear spatially well separated within amoeba trophozoites. L. pneumophila Paris (pink) and P. amoebophila (green) at 48 hpi are visualized as described above. The lack of overlap between L. pneumophila and P. amoebophila-specific probes indicates that both bacteria are located in distinct vacuoles. Note that P. amoebophila cells are enclosed individually in host-derived inclusions. Blue color indicates DNA (DAPI staining); scale bar, 5 μm. Download FIG S3, PDF file, 2.6 MB. Copyright © 2019 König et al. 2019 König et al. This content is distributed under the terms of the Creative Commons Attribution 4.0 International license.

Only when the symbionts were present did amoebae fully recover from the L. pneumophila infection after an incubation time of 5 weeks, documented by an increase in amoeba numbers that was similar to those of an unchallenged control, as shown for L. pneumophila Lp02-T (Fig. 1C and D and Table S1). Strikingly, at 5 wpi L. pneumophila Paris could not be detected in recovered amoebae, either by fluorescent *in situ* hybridization (FISH) or PCR, whereas symbiont-free amoebae were lysed or infected with L. pneumophila at this stage (Fig. 1B, E, and F). We noted that the L. pneumophila strain used as well as MOI and incubation temperature likely affect the degree of amoeba recovery: L. pneumophila Paris had a stronger negative effect on amoebae than Lp02-T under the same conditions (MOI of 20, 30°C); amoebae infected with Lp02-T over 5 weeks only fully recovered at 30°C but not 20°C, and at 1 week postinfection different L. pneumophila Paris MOIs affected amoeba numbers to various degrees (Table S1 and Fig. S5A). Of note, the chlamydial symbionts remained present throughout the experiment at similar levels under all conditions (Fig. 1E and F).

Taken together, a commonly used laboratory strain of free-living amoebae carrying the chlamydial symbiont P. amoebophila is resilient to infection with L. pneumophila, a human pathogen and amoeba parasite that typically exploits and lyses its host cells. Consequently, the symbionts confer direct or indirect protection that leads to reduced pathogen levels. Pathogen reduction sets in early during *Legionella* infection and may ultimately be responsible for amoeba recovery.

### Symbiont-mediated protection in freshly isolated environmental amoeba and L. pneumophila isolates.

Long-term axenic culture of *Acanthamoeba* isolates eventually leads to adaptation and altered traits, such as decreased temperature tolerance and reduced ability to encyst (50, 51). To account for this bias, we explored the relevance of our findings for amoeba freshly recovered from environmental samples. Two *Acanthamoeba* isolates (designated ML and 2HH), both belonging to the same sequence type (T4) as A. castellanii Neff, were first infected with P. amoebophila; once continuous symbiont-containing amoeba cultures were established, they were challenged with L. pneumophila. In addition to L. pneumophila strains Paris and Lp02-T, we also included two freshly obtained environmental L. pneumophila isolates (strains 3626/10 and 3621).

**FIG 2:**
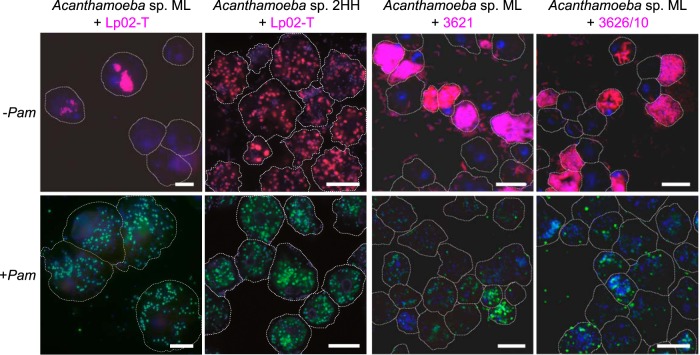
Environmental amoeba isolates harboring the symbiont P. amoebophila eliminate different L. pneumophila strains. FISH combined with DAPI staining (blue) was performed at 5 weeks after L. pneumophila infection (MOI of 20, 20°C). Amoeba and L. pneumophila strains used are indicated on the top of each set of images, in which the first row shows infections without the symbiont (−*Pam*) and the second row with the symbiont (+*Pam*). Initial infection with L. pneumophila was determined at 2 hpi (diamond symbols in Fig. 4). FISH probes specifically targeted L. pneumophila (LEGPNE1, magenta) and the chlamydial symbiont (Chls-523, green). Amoeba outlines are indicated by white dotted lines. Scale bars, 10 μm.

As observed for the amoeba laboratory strain, L. pneumophila numbers were reduced at the end of each experiment with environmental amoeba that contained P. amoebophila compared with those of the symbiont-free control (Table S1). Importantly, when the symbiont was present, both recent amoeba isolates could be completely cured from L. pneumophila Lp02-T infection 5 wpi at 20°C (Fig. 2 and Fig. S4 and S5B); likewise, both recent L. pneumophila strains were cleared from symbiont-harboring *Acanthamoeba* sp. strain ML (Fig. 2 and Table S1). Amoeba recovery, measured as amoeba net growth, was again observed only at 5 wpi (Table S1); the amoeba isolate *Acanthamoeba* sp. strain ML harboring the symbiont was even able to grow significantly better at both 20°C and 30°C (Fig. S5B). In contrast to the amoeba laboratory strain, however, symbiont-free amoeba numbers remained unchanged and even increased in one instance 5 weeks after L. pneumophila infection (Table S1). The differences in the extent of L. pneumophila inhibition and amoeba recovery observed at two incubation temperatures and between environmental amoeba isolates and the laboratory strain indicate that host and temperature contribute to the efficiency of symbiont-mediated inhibition of L. pneumophila.

10.1128/mBio.00333-19.6 FIG S4 Clearance of L. pneumophila in symbiont-containing amoebae as demonstrated by PCR. At 5 weeks post-L. pneumophila infection, DNA was extracted from replicate cultures with (+*Pam*) or without (−*Pam*) the chlamydial symbiont, and L. pneumophila was targeted by PCR primers specific for the L. pneumophila *mip* gene. In the case of infection of A. castellanii Neff with L. pneumophila Paris and *Acanthamoeba* sp. strain 2HH with L. pneumophila Lp02-T (left and center gel images), primers Legmip_f/Legmip_r were used (R. M. Ratcliff, J. A. Lanser, P. A. Manning, and M. W. Heuzenroeder, J Clin Microbiol **36:**1560–1567, 1998), whereas L. pneumophila Lp02-T infecting *Acanthamoeba* sp. strain ML was targeted using primers designed in this study (Lp02mipF/R, right gel image) (Text S1). All infection experiments were conducted at 20°C, with an MOI of 0.5 for Paris infecting Neff and an MOI of 20 for Lp02-T infecting 2HH and ML. M, marker; numbers 1 to 3, replicates; −, negative control; +, positive control. Download FIG S4, PDF file, 1.1 MB. Copyright © 2019 König et al. 2019 König et al. This content is distributed under the terms of the Creative Commons Attribution 4.0 International license.

10.1128/mBio.00333-19.7 FIG S5 Effect of L. pneumophila MOI and incubation temperature. (A) L. pneumophila MOI affects amoeba recovery. A. castellanii Neff cells with (+*Pam*) and without (−*Pam*) the symbiont were infected with L. pneumophila Paris using different MOIs (20°C), and amoeba numbers were determined one week postinfection. Note that in all experiments, amoeba numbers were reduced compared to starting numbers (658.7 ± 113.3 amoebae/μl), but the reduction occurred to various degrees depending on MOI and presence of the symbiont. Importantly, in all cases L. pneumophila numbers were lower when the symbiont was present (Table S1). At low (0.5) and high (10) L. pneumophila MOI, cell numbers of amoebae without the symbiont did not decline as much as those of amoebae with the symbiont, indicating that infection load (MOI) plays a role. In contrast, when MOIs of 2, 5, and 8 were used, amoebae without the symbiont declined to a greater extent than amoebae with the symbiont. The difference between amoebae with or without *Pam* was statistically significant for MOIs of 0.5, 5, 8, and 10 (*, *P* < 0.01, unpaired *t* test). Error bars mark the standard deviations from three biological replicates. (B) Temperature affects L. pneumophila infection level but not amoeba recovery. *Acanthamoeba* sp. strain ML amoebae with (+*Pam*) and without (−*Pam*) the symbiont were infected with L. pneumophila Lp02-T (MOI of 20). After 5 weeks of incubation at either 20°C or 30°C, the percentage of L. pneumophila-infected amoebae (upper) and amoeba growth (lower) were determined. L. pneumophila was only cleared at 20°C (*, *P* < 0.05 at 20°C, *P* = 0.14 at 30°C). As for the laboratory strain A. castellanii Neff, *Acanthamoeba* sp. strain ML harboring the symbiont showed growth over 5 weeks postinfection. Controls without the symbiont exhibited zero net growth, indicating a certain level of resilience even without the symbiont (*, *P* < 0.01 at both temperatures). Statistically significant differences were determined using the unpaired *t* test. (A and B) Error bars mark the standard deviations from three biological replicates. Download FIG S5, PDF file, 0.04 MB. Copyright © 2019 König et al. 2019 König et al. This content is distributed under the terms of the Creative Commons Attribution 4.0 International license.

Thus, in the face of infection with L. pneumophila, the presence of the symbiont P. amoebophila also provides an advantage for two environmental amoeba strains, even though *Acanthamoeba* sp. strain ML by itself is less susceptible to *L pneumophila* under the conditions applied in this study. Notably, symbionts also protected amoebae against two environmental L. pneumophila isolates. These findings indicate that symbiont-mediated protection plays a role in the natural environment.

Altogether, our results suggest that while the extent of resistance against L. pneumophila is likely influenced by the host strain, infectious load (MOI), and temperature, protection is provided if amoebae can sustain the chlamydial symbiont. Of note, we have never observed clearance of L. pneumophila in the absence of the symbiont. Protection therefore strictly relies on the presence of the chlamydial symbiont.

### Improved fitness of amoebae that recovered from L. pneumophila infection.

**FIG 3:**
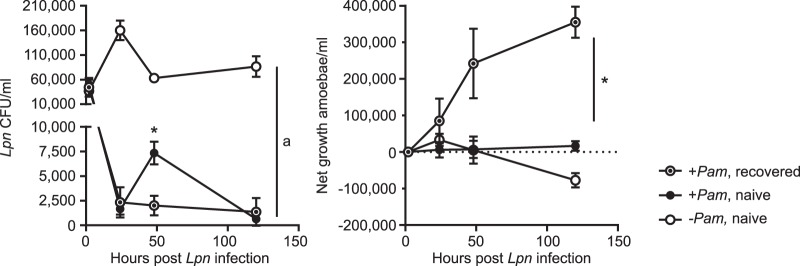
Increased fitness of a recovered amoeba isolate in the face of L. pneumophila Lp02-T infection. *Acanthamoeba* sp. strain ML previously not exposed to L. pneumophila Lp02-T (*Lpn*; amoebae termed naive) or exposed but cleared from L. pneumophila (termed recovered) were infected with L. pneumophila Lp02-T (MOI of 20, 20°C). Gentamicin treatment was performed to kill extracellular bacteria, and L. pneumophila numbers (left) and net amoeba growth (right) were determined, starting at 2 hpi. The presence and absence of endosymbionts are indicated by +*Pam* and −*Pam*, respectively. Error bars show standard deviations from three biological replicates. In total, L. pneumophila growth is only significantly different between symbiont-free amoebae and both conditions with the symbiont present (*, *P* < 0.05 by one-way ANOVA and Tukey's test). However, L. pneumophila numbers at 48 hpi also significantly vary between recovered and naive amoebae harboring the symbiont (*, *P* < 0.01 by unpaired *t* test). Amoeba growth is significantly different between recovered amoebae (+*Pam*) and naive amoebae with and without symbionts at the last two time points (*, *P* < 0.001 by two-way ANOVA and Tukey's test).

We next tested whether the fully recovered, L. pneumophila-cleared amoeba isolate from the previous infection experiment (*Acanthamoeba* sp. strain ML) was altered in terms of L. pneumophila susceptibility and amoeba growth when again exposed to the pathogen. We observed that L. pneumophila Lp02-T growth was strongly inhibited in symbiont-harboring amoebae independent of whether the amoebae were not exposed to L. pneumophila before (naive) or have recovered from a previous L. pneumophila infection (Fig. 3). However, consistent with our observations for A. castellanii Neff (Fig. S2B and S3), L. pneumophila cell numbers increased initially within naive symbiont-containing amoebae at 48 hpi. In contrast, in recovered amoebae L. pneumophila numbers decreased continuously (Fig. 3). This enhanced inhibition of pathogen growth in recovered amoebae entailed a remarkably increased growth compared to that of naive amoebae (Fig. 3). Consequently, the first exposure to the amoeba pathogen endowed the symbiont-harboring amoeba isolate with the capacity to more efficiently restrict L. pneumophila proliferation, promoting superior amoeba growth compared to that of naive amoebae.

### Symbiont-mediated defense is not caused by reduced host cell invasion.

The reduced number of L. pneumophila cells in symbiont-containing amoebae at the end of an infection experiment could be the consequence of impaired host cell invasion. While the mode of host cell entry of the P. amoebophila symbiont is still unknown, L. pneumophila uptake is facilitated by receptor-mediated endocytosis (52). Thus, either competition for or symbiont-stimulated downregulation of L. pneumophila receptors could decrease the rate of host cell invasion.

**FIG 4:**
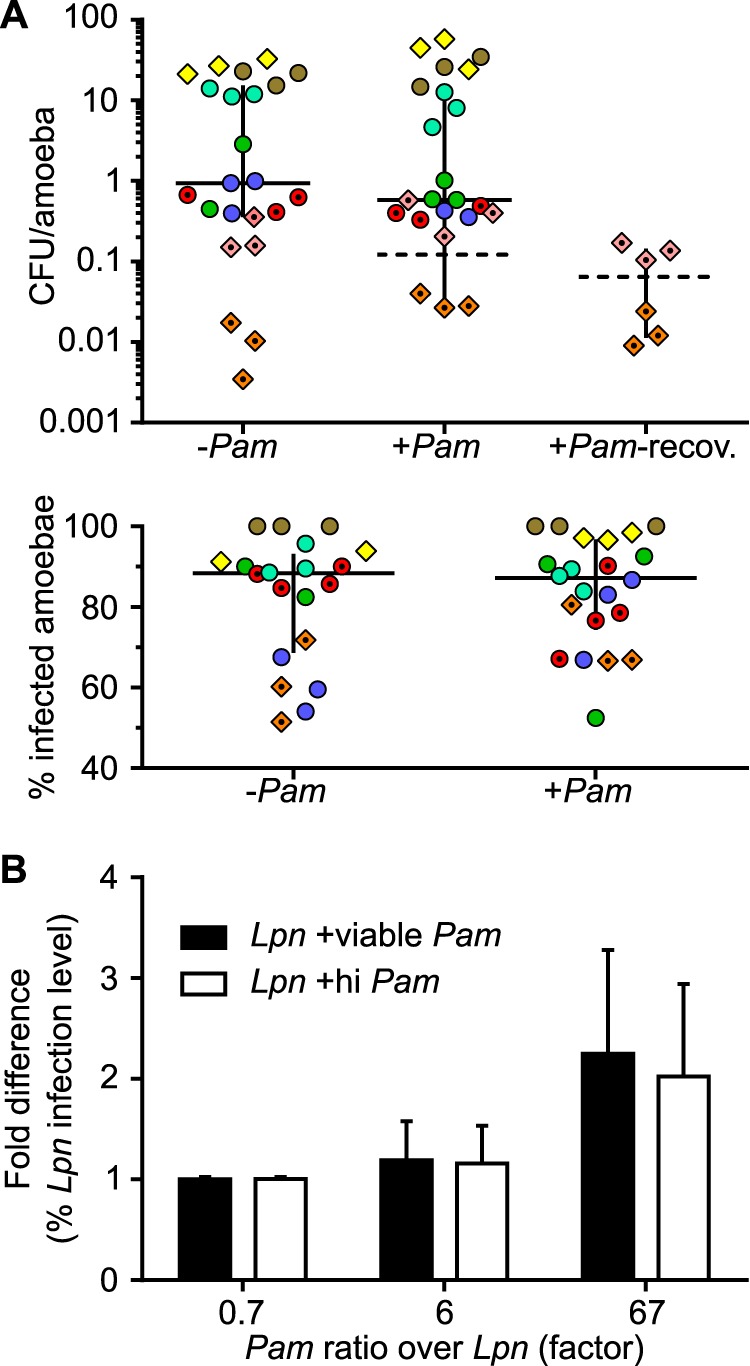
The symbiont P. amoebophila does not affect L. pneumophila uptake and host cell invasion. (A) To assess susceptibility of amoebae to L. pneumophila infection, we measured the number of viable L. pneumophila cells per amoeba cell (upper) and percentage of L. pneumophila-infected amoebae (lower), both at 2 hpi. CFU/amoeba were determined not only for naive amoebae with (+*Pam*) and without the symbiont (−*Pam*) but also for fully recovered symbiont-harboring amoebae (+*Pam*-recov.). Color groups denote separate experiments, with each data point representing a biological replicate. Circles show results from experiments using A. castellanii Neff, whereas experiments conducted with *Acanthamoeba* sp. strain ML are represented by diamonds. Data points filled with color were obtained using L. pneumophila Paris, and data points with black dots were obtained using L. pneumophila Lp02-T. Horizontal solid lines indicate medians, taking into account all data points. Dotted lines show medians only considering the infections with symbionts from Fig. 3 (pink and orange diamond shapes). Error bars denote interquartile ranges. No individual experiment yielded a statistically significant difference between the presence and absence of the symbiont or between naive and recovered amoebae (*P* > 0.05 by unpaired *t* test). (B) The role of extracellularly present symbionts in invasion by L. pneumophila was assessed by adding viable or heat-inactivated (hi) P. amoebophila (*Pam*) to uninfected A. castellanii Neff together with L. pneumophila Lp02-T (*Lpn*) but at different ratios. At 2 hpi, L. pneumophila infection levels were determined and compared to the respective levels for L. pneumophila only (control). A fold difference around 1 indicates that L. pneumophila infection levels were similar between treatment (viable or heat-inactivated P. amoebophila) and the control. The experiment was conducted in three biological replicates. Error bars indicate the 95% confidence intervals of a ratio of two means. Infections of A. castellanii Neff with L. pneumophila Lp02-T were carried out at 30°C; all other infections shown were conducted at 20°C.

To explore whether the presence of the symbiont within amoebae affects initial susceptibility of amoebae to L. pneumophila, we determined the number of L. pneumophila cells that could successfully infect amoebae, as well as the relative number of amoebae that were infected by L. pneumophila shortly after infection. Tested in numerous experiments (using different amoeba and L. pneumophila strains), we could not detect any significant differences in susceptibility, as amoebae both with and without the symbiont were invaded by comparable numbers of L. pneumophila (CFU/amoeba) and at similar frequency (percent infected amoebae) (Fig. 4A). Notably, the fully recovered, symbiont-harboring amoeba isolate *Acanthamoeba* sp. strain ML also did not show a significantly decreased susceptibility to reinfection with L. pneumophila Lp02-T compared with that of the naive counterparts (Fig. 4A, upper, orange diamonds). FISH performed at 2 hpi independently confirmed this similar invasion efficiency of two L. pneumophila strains (Fig. S3).

To further demonstrate that there is no uptake inhibition and/or receptor competition between P. amoebophila and L. pneumophila, we exploited the fact that the chlamydial symbionts are also transmitted horizontally and therefore also occur outside the host cell. If the bacteria used similar routes for host cell entry, extracellular symbionts in excess over L. pneumophila levels could hinder their uptake, eventually causing a delay in invasion by L. pneumophila. We tested this hypothesis by infecting symbiont-free A. castellanii Neff with different mixtures of viable or heat-inactivated P. amoebophila and infectious L. pneumophila Lp02-T. L. pneumophila infection levels at 2 hpi were then compared to those of controls in which only L. pneumophila was added. When the symbiont and L. pneumophila were approximately equally abundant, or when the symbiont was slightly more abundant than L. pneumophila (symbiont/pathogen ratio of 6:1), the symbionts did not affect the uptake of L. pneumophila (Fig. 4B). Unexpectedly, when the symbiont was added in greater excess over L. pneumophila (symbiont/pathogen ratio of 67:1), the proportion of L. pneumophila-infected amoebae was twice as high as that of the control without the symbiont (Fig. 4B). The addition of heat-inactivated symbionts had a similar effect on L. pneumophila uptake (Fig. 4B).

Altogether, neither symbionts present within the amoebae nor extracellular symbionts hampered invasion of amoebae by L. pneumophila. Instead, amoebae harboring symbionts were as susceptible to infection by the pathogen as symbiont-free amoebae. Moreover, the presence of a large number of extracellular symbionts appeared to stimulate uptake of L. pneumophila (independent from symbiont viability). The reduced L. pneumophila load we observed at 1 week postinfection (Fig. S2 and S3 and Table S1) is therefore not a result of constrained L. pneumophila uptake during initial infection stages. Also, reduced amoeba-to-amoeba transmission of L. pneumophila by extracellular symbionts potentially blocking uptake can be ruled out as a factor contributing to the observed symbiont-mediated protection. Thus, we postulate that the mechanism responsible for symbiont-mediated defense involves inhibiting intra-amoeba development of L. pneumophila, which ultimately could impair transmission of this pathogen.

### Decreased infectivity of L. pneumophila released from symbiont-harboring amoebae.

**FIG 5:**
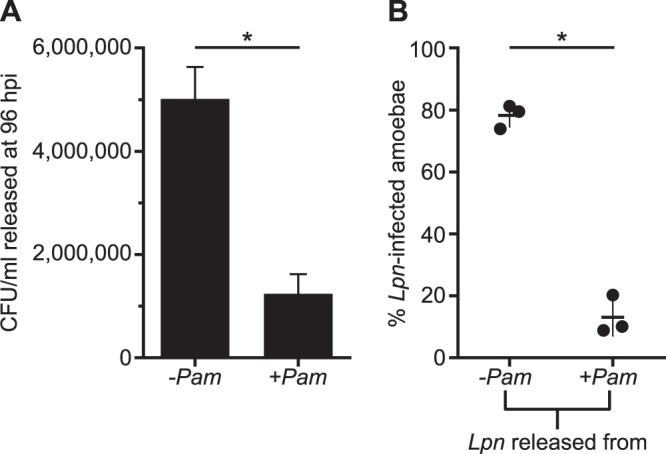
L. pneumophila released from symbiont-harboring amoebae are reduced in number and infectivity. L. pneumophila strain Paris was used to infect *Acanthamoeba* sp. strain ML (MOI of 5, 20°C). At 96 hpi, the supernatants containing the released L. pneumophila cells were harvested, and L. pneumophila cells were counted (A) and used to infect symbiont-free amoebae at an MOI of 30 (−*Pam*) or 23 (+*Pam*) (B). Both the CFU/ml released at 96 hpi and the percentage of amoebae at 2 hpi infected with L. pneumophila (DAPI counts) were significantly different from those of the control, even when normalized for the slight difference of a factor of 1.3 in MOI (*, *P* < 0.001 by unpaired *t* test). Error bars indicate standard deviations, and horizontal lines in panel B show the means from three biological replicates. –*Pam*, P. amoebophila absent; +*Pam*, P. amoebophila present.

If transmission of L. pneumophila was indeed impaired by an intra-amoeba, endosymbiont-dependent inhibition of L. pneumophila development, we would expect to observe a negative effect on replication, development, and/or release of L. pneumophila from symbiont-containing amoebae. To test this hypothesis, we quantified L. pneumophila Paris released from *Acanthamoeba* sp. strain ML into the supernatant at late infection stages and also determined their infectivity. We chose 96 hpi for collecting the supernatants, because light microscopic inspection of infected cultures indicated massive release of L. pneumophila from symbiont-free amoebae at this time point. By plating the supernatants, we indeed recorded a marked (4-fold) decrease in the number of L. pneumophila organisms released in the presence of the symbiont at this time point during infection (Fig. 5A), a ratio that could be confirmed when counting L. pneumophila via filtration and 4′,6-diamidino-2-phenylindole (DAPI) staining (data not shown). Based on L. pneumophila numbers determined by DAPI staining, symbiont-free amoebae were subsequently infected with equal numbers of L. pneumophila released from symbiont-containing and symbiont-free amoebae. Strikingly, the proportion of infected amoebae at 2 hpi was significantly lower when L. pneumophila originated from symbiont-harboring amoebae than from symbiont-free amoebae (Fig. 5B). This pronounced difference in infectivity indicates that L. pneumophila released from symbiont-harboring amoebae is less virulent than L. pneumophila released from symbiont-free amoebae.

Thus, the presence of the P. amoebophila symbiont either slows down or blocks the progression of the intracellular life cycle of L. pneumophila, resulting in a reduction of pathogen progeny. Together with the observed decrease of infectivity, these two effects may ultimately lead to elimination of L. pneumophila from the host amoebae population containing P. amoebophila, as observed in our experiments.

### Altered L. pneumophila and P. amoebophila gene expression during coinfection.

To better understand the impact of P. amoebophila on the life cycle of intracellular L. pneumophila, we analyzed the gene expression dynamics of both bacteria during single infection or coinfection. We determined gene expression levels by RNA sequencing (RNA-Seq) at 24 hpi and 96 hpi, corresponding to the replicative and transmissive phase, respectively, of L. pneumophila Paris in symbiont-free as well as in symbiont-containing A. castellanii Neff. At the later time point, intracellular bacteria as well as legionellae released from amoeba host cells were analyzed separately. Transcripts were detected for 82 to 98% of all genes for both L. pneumophila and P. amoebophila (Table S2). Differential gene expression analysis showed that up to 1,079 genes were significantly up- or downregulated between time points and depending on the presence/absence of the symbiont or pathogen (corresponding to 66% of all expressed genes) (Fig. S6 and Data set S1). To understand these pronounced changes, we determined functional categories and processes significantly overrepresented among the set of differentially expressed genes (Fig. S6).

10.1128/mBio.00333-19.2 TABLE S2 RNA-Seq read and mapping statistics. Download Table S2, DOCX file, 0.02 MB. Copyright © 2019 König et al. 2019 König et al. This content is distributed under the terms of the Creative Commons Attribution 4.0 International license.

10.1128/mBio.00333-19.8 FIG S6 Enriched functional categories and processes among differentially expressed P. amoebophila and L. pneumophila Paris genes. Gene expression was determined by RNA-Seq. All functional categories that were significantly overrepresented (false discovery rate, <0.05) among up (↑)- and downregulated (↓) genes are shown (for details see Data set S1). Numbers of up- and downregulated genes are depicted. Long arrows point out the direction of comparison, e.g., 450 symbiont genes were significantly upregulated at 24 h post-L. pneumophila infection (24 hpi) compared to the uninfected control (A), and 685 L. pneumophila genes were significantly downregulated at 96 hpi compared to levels at 24 hpi, when the amoebae did not harbor symbionts (B). (A) Terms in grey were found by trend to be enriched, as evaluated by manual inspection. Note that some genes encoding a putative type IV secretion system (*tra* genes) were also detected to be differentially expressed. (B) Terms in black are shared between the two conditions (amoebae with and without symbionts), and terms in blue are unique for the respective condition. The putative DNA transfer island known as *lvr* genes (grey) was significantly differentially expressed where indicated but failed to show up as overrepresented due to missing automatic functional annotation. T3SS, type 3 secretion system; LPS, lipopolysaccharide; Pro, proline; Met, methionine; Ala, alanine; Tyr, tyrosine; Lpn, L. pneumophila Paris; Pam, P. amoebophila. Download FIG S6, PDF file, 0.05 MB. Copyright © 2019 König et al. 2019 König et al. This content is distributed under the terms of the Creative Commons Attribution 4.0 International license.

10.1128/mBio.00333-19.9 DATA SET S1 Gene expression values, gene annotations, and enrichment analyses of P. amoebophila and L. pneumophila Paris. Download Data Set S1, XLSX file, 1.5 MB. Copyright © 2019 König et al. 2019 König et al. This content is distributed under the terms of the Creative Commons Attribution 4.0 International license.

At 24 hpi and in the presence of L. pneumophila, P. amoebophila upregulated a large number of stress response-related genes and genes encoding type 3 secretion system (T3SS) components and putative effector proteins. Conversely, processes such as translation, transcription, and amino acid and fatty acid metabolism were downregulated at this time point. At 96 hpi, the genes involved in metabolism were upregulated again and were comparable to gene expression levels observed in the absence of L. pneumophila, whereas T3SS-related genes were downregulated. Together, this mRNA profile suggests that at 24 hpi with L. pneumophila, the symbiont induces a general stress response and dramatically shuts down its metabolism and replication. The symbiont reacts to L. pneumophila infection by enhancing protein secretion, including a range of (new) effectors, indicating that P. amoebophila first struggles to maintain its intracellular niche and later adjusts to the changed environment by additional remodelling of host cellular processes. At 96 hpi the symbiont appears to have managed to take over host cell control again, and expression of metabolic genes is back to normal, i.e., resembles the situation without L. pneumophila.

**FIG 6:**
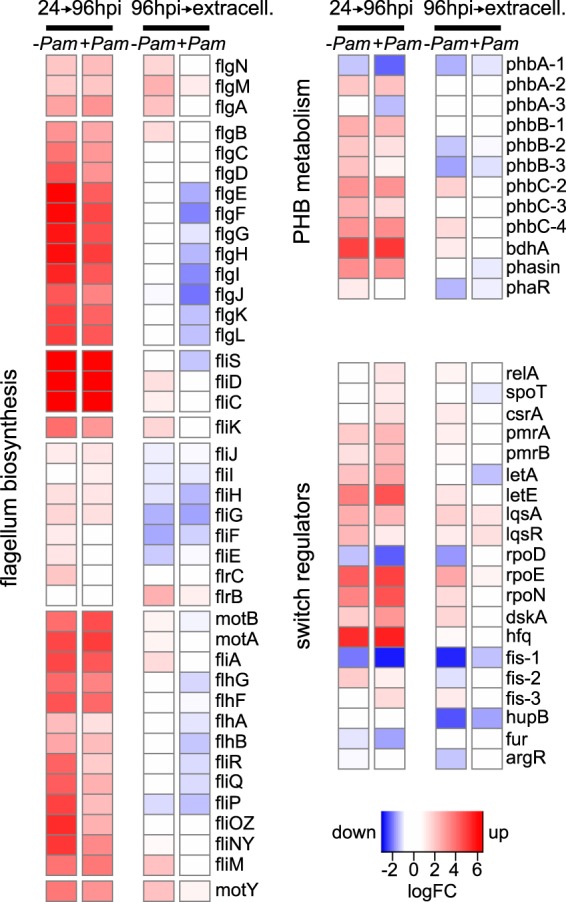
Altered L. pneumophila gene expression in the presence of the symbiont suggests impaired transition from replicative to transmissive phase. Transcriptomes of L. pneumophila Paris infecting A. castellanii Neff with (+*Pam*) and without (−*Pam*) the P. amoebophila symbiont at 24 h, at 96 h within amoebae, and after host cell release (extracell) were determined by RNA-Seq (20°C; for MOIs see Materials and Methods). Processes and functional categories that were significantly enriched among differentially expressed genes include flagellum biosynthesis, PHB metabolism, and genes involved in growth phase regulation (Fig. S6). Heatmaps show log_2_ fold changes (logFC) of all genes that were differentially expressed between at least one pair of conditions (false discovery rate of <0.05). A logFC below 1 (no differential expression) is shown in white, significant downregulation is shown in blue, and significant upregulation is shown in red. The stronger the color, the stronger the gene expression change between two conditions. Flagellar genes are ordered by genetic locus, and the other genes are ordered by processes. Note that *fleQ*, *phbC-1*, *letS*, *lqsS*, *lqsT*, *rpoS*, *cpxA*, and *cpxR* were not differentially expressed. 24→96hpi, expression at 96 hpi compared to that at 24 hpi; 96hpi→extracell., extracellular expression (96 hpi) compared to intracellular expression.

For L. pneumophila, the presence of the symbiont did not have a strong effect during early infection stages. Gene expression was not altered substantially at 24 hpi compared to the situation without P. amoebophila (5% differentially expressed genes only) (Fig. S6). However, there are a number of striking differences during the progression of the L. pneumophila life cycle. In the presence of the symbiont and contrary to the single infection, DNA replication, respiration, and glycolytic processes were not downregulated at 96 hpi, and genes involved in polyhydroxybutyrate (PHB) synthesis were not upregulated at this time point (Fig. 6 and Fig. S6). Similarly, while a range of regulatory genes, including the pivotal regulator *csrA* (53, 54), were upregulated in L. pneumophila cells released from the amoeba host, these genes remained unchanged in the presence of the symbiont (Fig. 6). Instead, ABC transporters, including import proteins for amino acids (the substrate for intracellular growth of legionellae) (89), remain highly expressed at 96 hpi, and a pronounced downregulation of genes responsible for flagellar assembly was observed when the symbiont was present (Fig. 6 and Fig. S6). This mRNA profile indicates that L. pneumophila infection starts normally despite the presence of the P. amoebophila symbiont. Consistent with the findings in our infection experiments, L. pneumophila is taken up and starts to replicate within amoeba cells (Fig. 4 and Fig. S2B and S3). However, at 96 hpi, which typically marks the end of the infection cycle, processes characteristic of the transmissive phase are impaired, including PHB metabolism and flagellum synthesis (55, 56). The lack of downregulation of metabolic functions and persistent expression of amino acid transporters indicate that L. pneumophila is still equipped to acquire nutrients long after the initial infection. These gene expression profiles in the presence of the symbiont are consistent with an obstructed transition to the transmissive phase and the release of replicative, noninfectious L. pneumophila cells, as observed in our infection experiments (Fig. 5).

## DISCUSSION

### Symbiont-mediated protection of amoebae.

**FIG 7:**
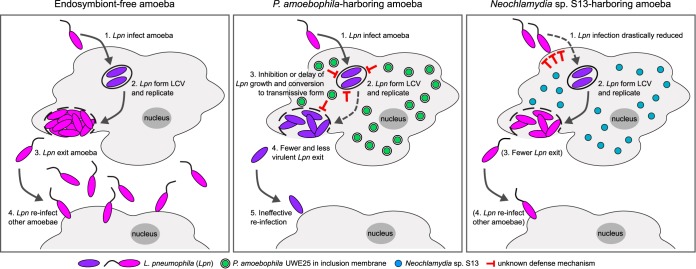
Symbiont-mediated defense against L. pneumophila. In the absence of chlamydial endosymbionts, L. pneumophila undergoes a characteristic intra-amoeba life cycle involving entry, replication within a *Legionella*-containing vacuole (LCV), transition to the transmissive form, amoeba lysis, and bacterial escape. The transmissive form can subsequently infect other host cells (left). The present study, together with that of Maita et al. (47), demonstrates that chlamydial endosymbionts of *Acanthamoeba* spp. provide the host with protection against different strains of L. pneumophila, although the modes of protection are different. While *Neochlamydia* species-harboring amoebae block the uptake of L. pneumophila (right) (47), P. amoebophila-containing amoebae interfere with the intracellular L. pneumophila life cycle, resulting in a significantly reduced number of released bacteria that are less virulent (center) (this study). Steps 3 and 4 in the *Neochlamydia* sp. strain S13 model have not been demonstrated but would be expected to be a consequence of impaired L. pneumophila uptake (in parentheses). Note that the two types of endosymbionts differ in that P. amoebophila is enclosed within an inclusion membrane, whereas *Neochlamydia* sp. strain S13 is found directly in the host cytoplasm. Replicative-phase L. pneumophila is shown in dark violet, whereas transmissive forms are depicted in pink.

Animal-bacterium interactions are manifold and fundamentally impact animal evolution, development, biology, and ecology (57). Bacterial symbionts often provide nutrients to and recycle waste products from the host organism, and some may manipulate host reproduction (58, 59). In particular, insects also harbor bacterial symbionts that provide them with protection against natural enemies such as parasitic wasps, pathogenic fungi, and viruses (60–62). Symbiont-mediated defense is also an important role of complex animal microbiomes (63) but until recently was not known to extend to protists harboring symbionts (64, 65). Our study demonstrates that (i) in the presence of the chlamydial symbiont P. amoebophila, *Acanthamoeba* hosts survive infection by the amoeba parasite and human pathogen L. pneumophila, (ii) the mode of protection in this protist host involves failed formation of infectious transmissive L. pneumophila, and (iii) symbiont-mediated defense is a trait of both environmental and clinical isolates of amoebae and L. pneumophila. Together with recent findings on a related amoeba endosymbiont (46), we bring forward compelling evidence that chlamydial symbionts associated with free-living amoebae represent mutually beneficial symbioses with the host, providing nutrition and a sheltered environment, and with the symbionts, providing defense against parasite infection (Fig. 7).

### Modes of symbiont-mediated defense against L. pneumophila.

Interestingly, the mechanism of host protection differs for two chlamydial endosymbionts. Whereas amoebae harboring *Neochlamydia* sp. strain S13 exhibit severely reduced L. pneumophila entry caused by impaired phagocytosis (47), our data consistently show that P. amoebophila has no effect on host cell invasion by L. pneumophila (Fig. 4A). Instead, the presence of the symbiont likely perturbs intra-amoeba development of the pathogen, as impaired formation of fully virulent L. pneumophila Paris was indicated by two complementary experiments (Fig. 5 and 6; see also Fig. S6 in the supplemental material). These fundamentally different protection modes may seem surprising, as *Protochlamydia* and *Neochlamydia* both occur naturally as symbionts in amoebae. However, they are members of two related genera, and two important distinctions could account for their different protection mechanisms. First, P. amoebophila resides within host-derived membranes termed inclusions (66), whereas *Neochlamydia* sp. strain S13 can be found directly in the amoeba cytoplasm (35). This different level of cellular integration likely affects interaction with the amoeba host and may thus result in a fundamentally different host response to L. pneumophila infection. Second, different sets of chlamydial effector proteins delivered via type 2 and type 3 secretion systems could differentially modulate protection, as a large number of putatively secreted proteins unique for each symbiont has been identified (40, 46).

### Interference with the transition of L. pneumophila to the transmissive form.

Collectively, our data indicate that the presence of P. amoebophila leads to reduced L. pneumophila growth and an incomplete transition to the infectious transmissive stage. Monitoring the course of infection by FISH and plate counts for two L. pneumophila strains and two A. castellanii strains revealed that L. pneumophila uptake is generally not inhibited, and that the bacteria also multiply in the presence of the symbionts (Fig. 3 and 4 and Fig. S2 and S3). However, given the reduced overall number of viable L. pneumophila organisms 1 week postinfection, as well as the lower number of released bacteria 4 days postinfection than that of the symbiont-free control, L. pneumophila growth is either inhibited after a few rounds of replication or generally slowed down under the influence of the symbionts. This inhibition or delay of L. pneumophila development subsequently interferes with the pathogen’s conversion from the replicative to the transmissive stage (67), as substantial differences in gene expression indicate the lack of features required at the transmissive stage, including storage compound synthesis and a complete flagellar apparatus (Fig. 6). As a consequence, fewer and less infectious L. pneumophila cells are released from amoebae with symbionts (Fig. 5). Taking these findings together, L. pneumophila infecting P. amoebophila-harboring amoebae are targeted at an intracellular stage at which both growth and completion of the life cycle are impaired.

### Towards a molecular mechanism.

The exact molecular mechanism by which L. pneumophila infection is controlled in the presence of the chlamydial endosymbionts remains unknown for both P. amoebophila and *Neochlamydia* sp. strain S13 (47). In this study, we observed inhibition of L. pneumophila independent of the strain used; thus, the protection mechanism conferred by P. amoebophila is likely targeted against a conserved L. pneumophila feature. Irrespective of the specific target, different scenarios, or a mixture thereof, could explain the intracellular inhibition of L. pneumophila.

Some defensive microbes protect their host by interference competition, in which a toxin produced by the symbiont directly targets the parasite, pathogen, or predator (61, 65). The P. amoebophila genome does indeed encode proteins with classical polymorphic toxin domains, some of which are involved in interbacterial competition (68). A number of additional uncharacterized putative effector proteins secreted by the T3SS are also upregulated in the presence of L. pneumophila (Fig. S6). It is currently unclear, however, whether any of those have the potential to directly target and interfere with L. pneumophila development.

Alternatively, an indirect mode of defense could involve host immunity factors upregulated in response to the symbiont. Stimulated antimicrobial responses, some of which have been identified in the A. castellanii Neff genome (20, 69), could in turn act against L. pneumophila. For example, antimicrobial peptides are well-established mediators of innate immunity in eukaryotes that are known to be produced in response to symbionts (70). Autophagy can also act in pathogen clearance (71). Host immune mediation is well characterized for the endosymbiont *Wolbachia*, which induces a reactive oxygen species-dependent immune pathway that inhibits dengue virus proliferation in mosquito hosts (65, 72). Antimicrobial factors stimulated by P. amoebophila may be specific for L. pneumophila. Alternatively, the symbiont may protect itself against a general host antibacterial activity. The pronounced stress response of P. amoebophila upon L. pneumophila infection and the concomitant increased T3SS activity by the symbiont (including upregulation of both structural genes encoding the T3SS apparatus as well as novel putative effector proteins with eukaryotic-like domains) suggest that symbiont-induced modulation of host cellular pathways contributes to restricting growth and differentiation of L. pneumophila in amoeba. Because inhibition of L. pneumophila was recorded for three different *Acanthamoeba* host strains, conserved amoeba factors would play a role in this scenario.

Although each of the above-described scenarios remain plausible, we favor the model that the anti-L. pneumophila effect involves resource competition, as nutrients are tightly sequestered and thus may become scarce in the amoeba cytosol (73, 74). In this scenario, the obligate chlamydial symbiont would be better adapted to scavenge nutrients from the host than the facultative intracellular L. pneumophila and thus would severely restrict the resources needed by the invading pathogen to proliferate and differentiate to the infectious, transmissive form. Considering that P. amoebophila and L. pneumophila overlap in their nutrient requirements (e.g., glucose and amino acids) (39, 41, 89), L. pneumophila may indeed be starved for certain metabolites. Moreover, marked changes in the expression of transport and metabolism-related genes in both bacteria indicate that competition for resources occurs during coinfection and contributes to L. pneumophila inhibition. In fact, it is known that L. pneumophila differentiation and replication are governed by metabolic cues (67), and consequently a shortage or imbalance caused by the symbiont could perturb the intra-amoeba life cycle of L. pneumophila.

### The defensive response exerts a long-lasting effect.

Remarkably, past infection with L. pneumophila Lp02-T left an imprint on an environmental amoeba isolate that survived through symbiont-mediated protection. Like naive symbiont-harboring amoebae, amoebae that fully recovered from L. pneumophila infection were not more invasion resistant (Fig. 3 and 4A). However, fully recovered amoeba progeny did exhibit a more potent protection, as judged by L. pneumophila growth inhibition coupled with improved amoeba growth rate, suggesting that the previous encounter with L. pneumophila led to altered traits of the symbiont-amoeba system facilitating an even more powerful defensive response. Future studies can investigate whether the enhanced amoeba fitness is a general consequence of amoeba recovery and whether it is due to selection for symbiont-harboring amoebae equipped with stronger protective traits or instead an adaptation resembling a type of immunological memory.

### Conclusions.

Free-living amoebae with and without endosymbionts and L. pneumophila live in the same natural environments, such as biofilms in aquatic systems (3, 10). Maintaining chlamydial symbionts frequently comes at the cost of slower amoeba growth (Fig. S1). However, our study suggests that the symbionts equip the amoebae with an epigenetic-like defense, which provides a net fitness benefit when L. pneumophila is present (75). This defense is different from intrinsic amoeba antimicrobial defense strategies. It is both heritable and transferable, because the symbionts are transmitted vertically and horizontally. As symbiont-free amoebae are killed by pathogen-induced lysis, L. pneumophila represents a selective pressure expected to shape symbiont frequencies. Conversely, our data suggest that the presence of amoeba endosymbionts (in 25 to 100% of amoeba isolates; 13, 36) contributes to regulating abundance and virulence of L. pneumophila in the environment. Chlamydial symbionts of protists might be an important factor for the ecology of L. pneumophila and impact their capacity to cause opportunistic infections of humans.

## MATERIALS AND METHODS

### Bacteria and protist cultures.

The widely used laboratory strain Acanthamoeba castellanii Neff (ATCC 50373), with or without the endosymbiont Protochlamydia amoebophila UWE25 (ATCC PRA-7), was maintained in cell culture flasks (Nalge Nunc International, Rochester, NY, USA) at 20°C or 30°C in PYG medium (20 g/liter proteose peptone, 100 mM glucose, 2 g/liter yeast extract, 1 g/liter sodium citrate dihydrate, 4 mM MgSO_4_·7H_2_O, 1.32 mM Na_2_HPO_4_·2H_2_O, 2.5 mM KH_2_PO_4_, 0.05 mM Fe(NH_4_)_2_(SO_4_)_2_·6H_2_O; pH 6.5). Continuous cultures (i.e., asynchronous and when containing the endosymbiont, 100% infected) were regularly screened for contamination by fluorescence *in situ* hybridization (FISH) targeting most bacteria (probe mix of EUB338, EUB338 II, and EUB338 III; 76, 77) and DAPI staining (0.1 μg/ml in double-distilled water for 5 min).

*Acanthamoeba* sp. strain ML was recently isolated from sediment sampled from the Mono Lake in California. An axenic culture was obtained as described previously (36) and then confirmed to be symbiont free by FISH (mix of EUB338, EUB338 II, and EUB338 III) and DAPI staining. Sequencing of the 18S rRNA gene using the JDP primer set (78) assigned this new isolate to the most commonly isolated *Acanthamoeba* lineage, the T4 sequence type, which also includes A. castellanii Neff (79). *Acanthamoeba* sp. strain 2HH was isolated from patients who had developed a severe keratitis and were also found to be symbiont free and belonging to the T4 genotype (80). Both environmental isolates were infected with P. amoebophila as described below and maintained in culture as described above for A. castellanii Neff.

Legionella pneumophila Lp02 *thyA*^+^, a serogroup 1 Philadelphia-1 strain (here named L. pneumophila Lp02-T) that was converted back to thymidine prototrophy to enable infection of amoebae (81), and L. pneumophila strain Paris (CIP 107629T) (87), another serogroup 1 strain, were cultivated on *N*-(2-acetamido)-2-aminoethanesulfonic acid (ACES)-buffered charcoal yeast extract (CYE) plates and ACES-buffered yeast extract (AYE) broth, both at 37°C and supplemented with 0.4 g/liter l-cysteine and 0.135 g/liter ferric nitrate. L. pneumophila Lp02-T was additionally inoculated with 0.1 g/liter thymidine. Broth cultures were agitated on a roller drum (Eppendorf, Hamburg, Germany). CYE plates were prepared by addition of 2 g/liter charcoal and 15 g/liter agar to AYE broth. Two environmental L. pneumophila strains, 3621 and 3626/10 (both serogroup 1), were recently isolated from an unspecified Viennese water source and generously provided by the Austrian Agency for Health and Food Safety (AGES). They were cultivated like L. pneumophila Lp02-T. Small subunit (16S) rRNA and *mip* gene sequences were identical among all used L. pneumophila strains.

### FISH.

Aliquots of amoeba cultures were harvested and washed once with Page’s amoebic saline (0.12 g/liter NaCl, 0.004 g/liter MgSO_4_·7H_2_O, 0.004 g/liter CaCl_2_·2H_2_O, 0.142 g/liter Na_2_HPO_4_, 0.136 g/liter KH_2_PO_4_). Amoeba trophozoites were allowed to attach for 30 min on microscope glass slides with reaction wells (Marienfeld, Lauda-Königshofen, Germany) and then fixed with 4% paraformaldehyde for 10 min at room temperature. FISH was performed using the protocol, hybridization, and washing buffer described elsewhere (82). Briefly, fixed cells were washed with double-distilled water, and samples were dehydrated by incubation in increasing concentrations of ethanol (50%, 80%, and 96% for 3 min each), hybridized with respective Cy3, Cy5 (30 ng/μl), or FLUOS-labeled (50 ng/μl) rRNA-targeted oligonucleotide probes in hybridization buffer (0.9 M NaCl, 20 mM Tris-HCl, pH 8.0, 0.01% [wt/vol] SDS, 25% [vol/vol] formamide) for 1.5 h at 46°C in a hybridization chamber, and washed with prewarmed washing buffer (20 mM Tris-HCl, pH 8.0, 5 mM EDTA, pH 8.0, 149 mM NaCl) for 10 min at 48°C in a water bath, followed by a quick dip into ice-cold double-distilled water and drying using compressed air.

### Transfer of P. amoebophila to fresh amoeba isolates.

The supernatants of continuous A. castellanii Neff-P. amoebophila cultures containing released symbionts were harvested, cells were collected by centrifugation (10,620 × *g*, 15 min), and the suspension was filtered through 5-μm and 1.2-μm syringe filters (Sartorius, Göttingen, Germany) to remove residual amoeba cells. To make sure the original host was not cotransferred, the suspension was additionally freeze/thawed (−80°C/48°C) and subsequently vortexed with half of the volume of glass beads (diameter, 0.75 to 1 mm; Carl Roth, Karlsruhe, Germany). The cell debris was removed by centrifugation (150 × *g*, 10 min), and the supernatant was centrifuged (10,620 × *g*, 10 min) to collect the bacterial cells, after which they were used to inoculate PYG medium to check for remaining viable amoeba (control) or added to cultures of *Acanthamoeba* sp. strain ML and *Acanthamoeba* sp. strain 2HH.

### Preparation of L. pneumophila for infection.

AYE medium was inoculated with respective L. pneumophila strains, grown overnight at 37°C on a roller drum, diluted with fresh medium to an optical density at 600 nm (OD_600_) of 0.2 to 0.3, and grown to postexponential phase (OD_600_ of ≥3.5). Only cultures exhibiting a high proportion of motile cells were used for infection. Motility was assessed qualitatively as described previously (83). The number of viable L. pneumophila organisms added to amoebae was determined by diluting the cultures in infection buffer (84) and subsequent plating of dilutions on CYE plates in triplicate.

### Infection experiments to assess the protection effect.

One day prior to infection, cultures of symbiont-free amoebae and amoebae containing P. amoebophila were harvested, and multiwell plates or 25-cm^2^ culture flasks (Nalge Nunc) containing PYG medium were inoculated at densities allowing for comparable cell numbers between conditions and experiments on the day of infection. Different amoeba strains with and without symbionts were infected with different L. pneumophila strains at various multiplicities of infection (MOIs), either at 20°C or 30°C (see Table S1 in the supplemental material). To evaluate both short-term and long-term effects of exposure to L. pneumophila, infection experiments were conducted over 5 to 7 days or 5 weeks (Table S1). At 2 hpi, cocultures were washed three times with infection buffer to synchronize the infection, buffer was replaced with PYG medium, and cells were either harvested to assess starting levels of amoebae and L. pneumophila (see below) or further incubated at respective temperatures. Infections surveyed for up to 1 week continued to be harvested daily. In long-term infections, medium was replaced weekly, either by exchanging the medium or harvesting of cells, collection by centrifugation (6,800 × *g*, 5 min, room temperature), and transfer to fresh medium (Table S1). Long-term infections were evaluated 5 wpi by quantifying amoebae and L. pneumophila counts and, in some instances, by PCR (Table S1 and Text S1). Infection experiments were conducted in biological triplicate. To visualize infections, FISH was performed using probes detecting L. pneumophila (LEGPNE1) (85), chlamydiae (Chls-0523) (88), and amoebae (EUK516) (76), and images were taken using a confocal laser scanning microscope (510 Meta; Carl Zeiss, Jena, Germany; or TCS SP8; Leica, Wetzlar, Germany) or charge-coupled device camera (AxioCam HRc; Carl Zeiss) connected to an epifluorescence microscope (Axioplan 2 imaging; Carl Zeiss).

### Entry competition experiment.

Extracellular P. amoebophila organisms were freshly harvested from continuous A. castellanii Neff-P. amoebophila cultures as described above. After filtration, bacterial cells were collected by centrifugation (12,850 × *g*, 20 min, 4°C) and resuspended in infection buffer, and a small aliquot was counted by filtration onto polycarbonate membranes and subsequent DAPI staining as described previously (41). Bacterial suspensions were then split into two aliquots. One aliquot was heat inactivated for 1 h at 95°C and served as a dead control, whereas the other, containing viable P. amoebophila, was directly used. Symbiont-free A. castellanii Neff seeded into multiwell plates containing PYG medium was subsequently exposed to mixtures of either viable or heat-inactivated P. amoebophila with infectious L. pneumophila Lp02-T at three different ratios (L. pneumophila/P. amoebophila ratio, 1:0.7, 1:6, and 1:67) but keeping total numbers of bacterial cells and volumes constant. L. pneumophila without P. amoebophila served as the positive control; numbers added to the amoebae were equal to the numbers of L. pneumophila in the different mixtures. All treatments were conducted in biological triplicate. Infected amoebae were incubated at 30°C for 2 h and then harvested and fixed for FISH to determine the fraction of L. pneumophila-infected amoebae. Results are expressed as ratios between mean infection levels of mixtures and the control.

### Quantification and infectivity of released L. pneumophila.

*Acanthamoeba* sp. strain ML cultures with and without symbionts growing in PYG medium at 20°C were infected with L. pneumophila Paris (MOI of 5) in triplicate. At 2 hpi, infections were synchronized by washing four times with infection buffer, and aliquots were harvested to examine the percentage of infected amoebae by FISH. At 96 hpi, culture supernatants were harvested, filtered through 5-μm and 1.2-μm syringe filters to remove amoebae, and subsequently plated on CYE plates to determine CFU per ml of released L. pneumophila. To assess infectivity, replicate filtered supernatants were pooled, and cells were collected (8,300 × *g*, 10 min, room temperature) and counted as described above for P. amoebophila. Based on these counts, symbiont-free amoebae were infected in triplicate with equal numbers of released L. pneumophila cells. Supernatants were also plated on CYE plates to determine MOIs based on the number of viable L. pneumophila cells (L. pneumophila released from symbiont-free amoebae, MOI of 30; L. pneumophila released from symbiont-harboring amoebae, MOI of 23). After washing four times at 2 hpi, cells were harvested and fixed for FISH and DAPI staining, and the fraction of L. pneumophila-infected amoebae was determined.

### Infection of recovered amoebae.

Recovered symbiont-harboring *Acanthamoeba* sp. strain ML cells that were harvested 5 weeks after infection with L. pneumophila Lp02-T were seeded into PYG-containing multiwell plates. As a control, previously unexposed (naive) amoebae with and without the symbiont were seeded at equal densities. When grown to confluence at 20°C, amoebae were infected with L. pneumophila Lp02-T at an MOI of 20. The infection was synchronized by killing extracellular L. pneumophila with gentamicin (100 μg/ml) that was added 1 hpi. After an hour of incubation, gentamicin was removed by two washing steps with PYG medium. At 2, 24, 48, and 120 hpi at 20°C, cocultures were harvested to quantify amoebae and L. pneumophila.

### Quantification of amoebae, L. pneumophila, and L. pneumophila infection level.

To determine amoeba numbers per volume, amoebae were harvested by physically detaching amoebae from the culture surface at the indicated time points and directly counting cells using a Neubauer counting chamber. Amoeba growth is expressed as the difference between starting and final cell concentration (net growth). Amoeba cell sizes were determined using FISH images and the open-source image analysis software ImageJ (86). To monitor numbers of viable L. pneumophila, amoeba cocultures were harvested at different times postinfection, cells were collected by centrifugation (6,800 × *g*, 8 min, room temperature), pellets were resuspended in infection buffer, and amoebae were lysed by one freeze-thaw cycle (−20°C/48°C), followed by five passages through 26-gauge injection needles (B. Braun, Melsungen, Germany). Lysates were then plated at different dilutions on CYE plates to determine CFU/ml. Infection levels were determined by DAPI staining combined with FISH applying the L. pneumophila-specific probe (see above) and subsequent counting of infected relative to uninfected amoebae using an epifluorescence microscope. If necessary, infection levels were further classified as either low (1 to 5 bacteria/amoeba) or high (>5 bacteria/amoeba). Infection levels are expressed as percent L. pneumophila-infected amoebae.

### Transcriptome sequencing (RNA-Seq).

Symbiont-free as well as symbiont-containing cultures of A. castellanii Neff amoebae were harvested 3 days before infection, and 9 × 10^6^ amoebae per culture flask per time point were seeded in PYG medium and incubated at 20°C. Infectious L. pneumophila Paris cells were prepared as described above. Cultures were infected by adding L. pneumophila directly to the flasks, using an MOI of 5 for harvesting at 24 hpi and an MOI of 3 for harvesting at 96 hpi. Infected symbiont-free and symbiont-containing cultures, as well as uninfected symbiont-containing cultures, were then incubated for 2 h before amoebae were washed four times with infection buffer. PYG medium was added, and cultures were sampled at 2 hpi to monitor initial infection efficiency by FISH as described above and were further incubated at 20°C for 24 and 96 h. Released, extracellular L. pneumophila cells were collected at 96 hpi by centrifuging the supernatant of infected cultures at 150 × *g* for 2 min to roughly separate amoebae from bacterial cells, filtering the supernatant through 5-μm syringe filters (Sartorius) to remove residual amoebae, and finally pelleting the bacterial cells at 12,850 × *g* for 2 min. L. pneumophila-infected amoebae as well as uninfected amoebae were harvested, and bacteria were roughly enriched as previously described (41), but to optimize the yield of intracellular bacteria, cell suspensions were additionally vortexed for 1 min together with smaller glass beads (diameter, 0.25 to 0.5 mm; Carl Roth) after vortexing with larger beads (diameter, 0.75 to 1 mm; Carl Roth). Total RNA from extracellular bacteria (with and without symbionts) and intracellular bacteria enriched from cocultures at 24 and 96 hpi (with and without symbionts) was extracted using TRIzol reagent (Thermo Fisher Scientific, Waltham, MA, USA), and residual DNA was digested using the Turbo DNA-free kit (Thermo Fisher Scientific), both as described before (41). rRNA depletion using the Ribo-Zero gold rRNA removal kit (Illumina, San Diego, CA, USA), library preparation using the NEBNext Ultra RNA Library Prep kit for Illumina (New England Biolabs, Ipswich, MA, USA), as well as sequencing on an Illumina HiSeq 2500 with 100-bp read length was performed by the Vienna Biocenter Core Facilities (VBCF) Next-Generation Sequencing (NGS) Unit (http://www.vbcf.ac.at).

### Transcriptome analysis.

Sequencing reads were subjected to a cleaning workflow, reads were mapped to the P. amoebophila UWE25 (NC_005861.1) and L. pneumophila Paris (NC_006368.1) genomes, respectively, differential gene expression was determined, and statistically overrepresented functional categories were identified, all done as previously described (41). All samples were obtained from infection experiments set up in biological triplicate. L. pneumophila reads from one replicate at 96 hpi without symbionts were excluded from further analysis because the expression profile did not match those of the other replicates. Samples from uninfected symbiont-containing cultures were recovered from biological duplicates at both time points (24 and 96 hpi) but were treated as four replicates in gene expression analysis because of their nearly identical expression profiles. Detailed read and mapping statistics can be found in Table S2.

### Data availability.

*Acanthamoeba* sp. strain ML partial 18S rRNA gene sequence was deposited at GenBank and is accessible through accession number MH675534. RNA-Seq data are available at the Gene Expression Omnibus (GEO) database and are accessible through accession number GSE125876 (https://www.ncbi.nlm.nih.gov/geo/query/acc.cgi?acc=GSE125876).
