# Supplementary figures and images for: Symbiont-Mediated Defense against Legionella pneumophila in Amoebae
Source: mBio. 2019 May 14;10(3):e00333-19. doi: 10.1128/mBio.00333-19 (PMC6520448; doi:10.1128/mBio.00333-19)

**A**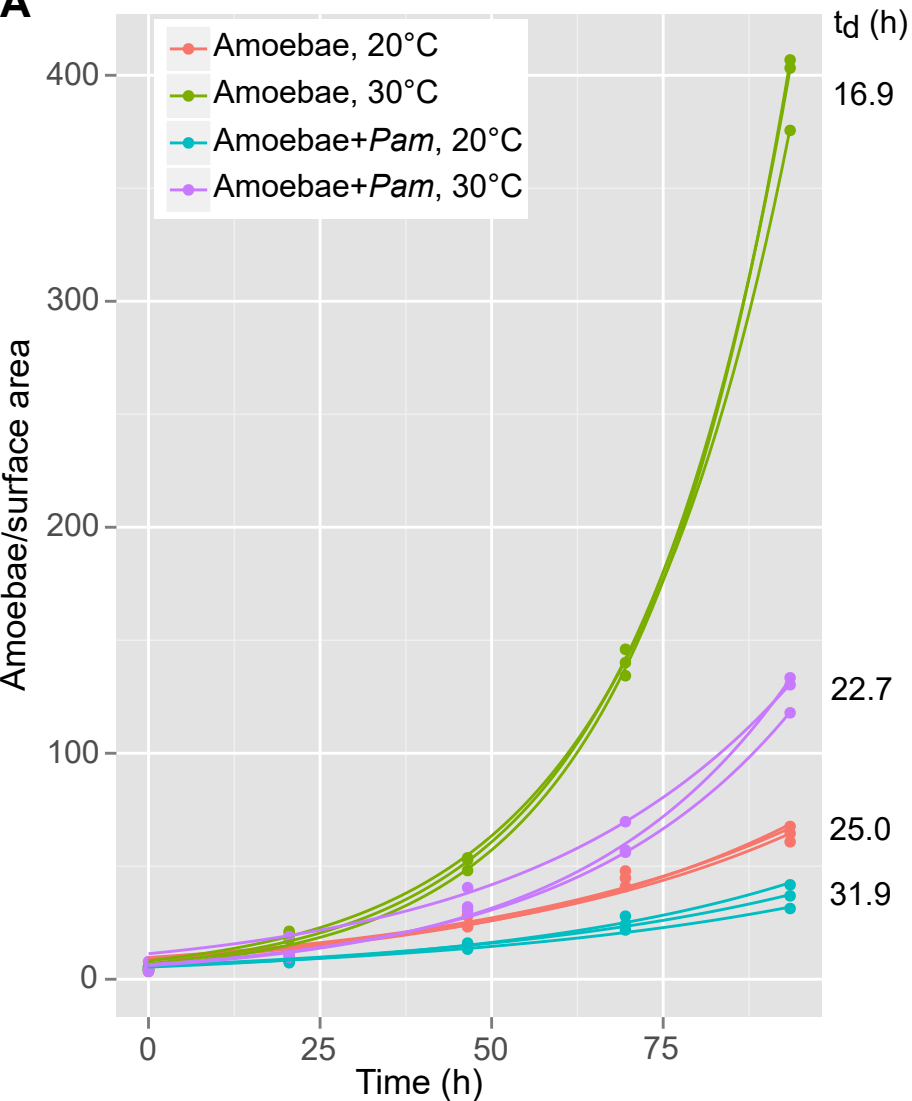**B**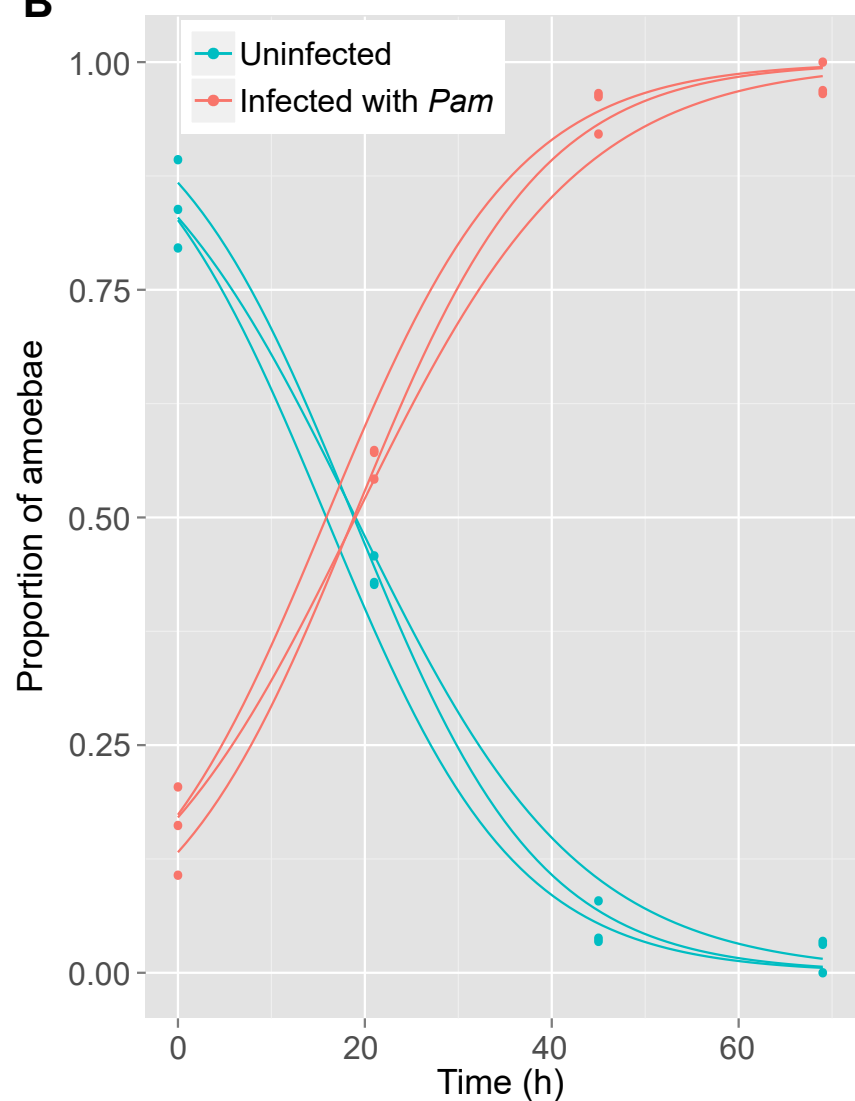

Supplement: FIG S1 [file mBio.00333-19-sf001.pdf]

A

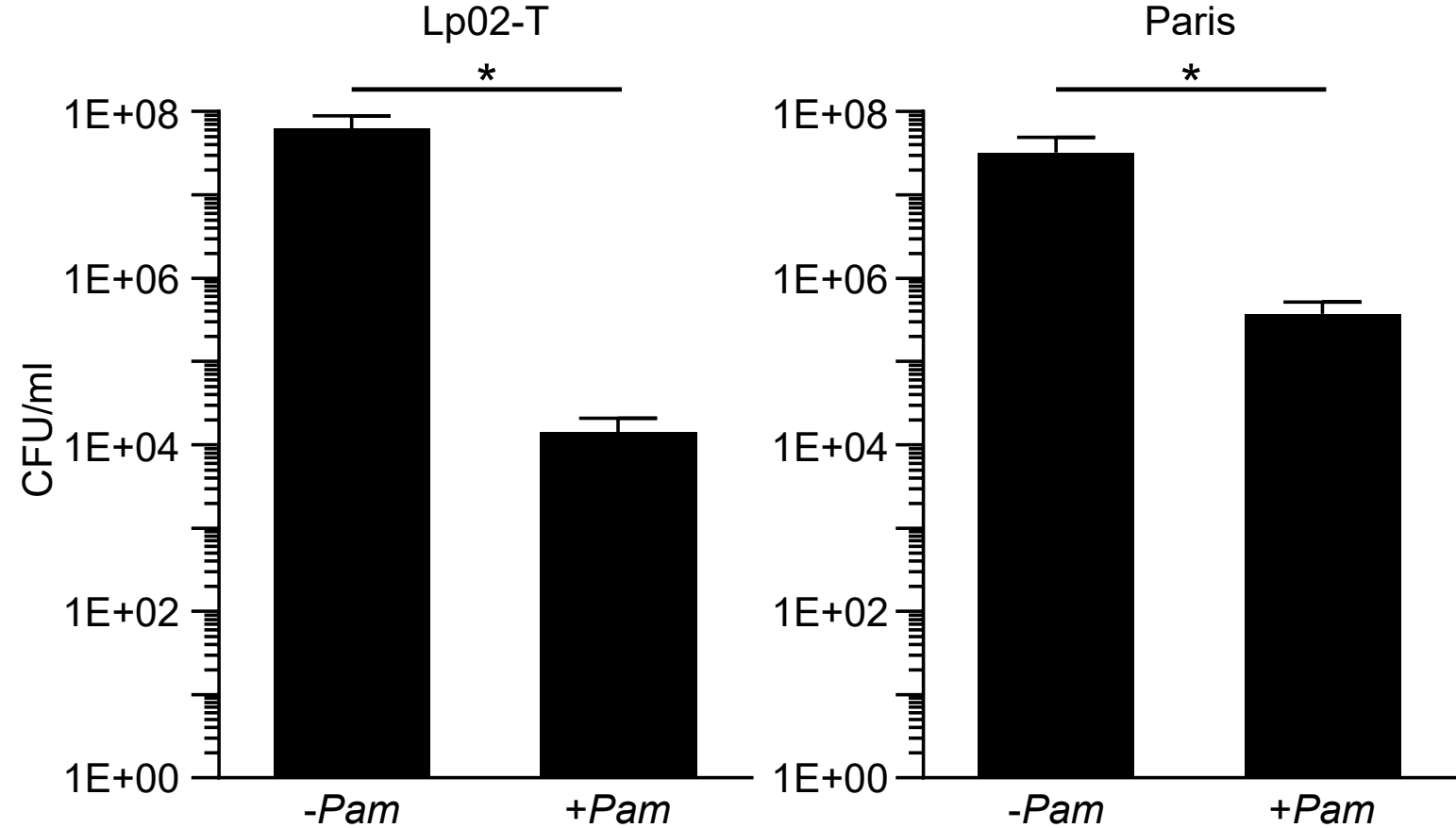

B

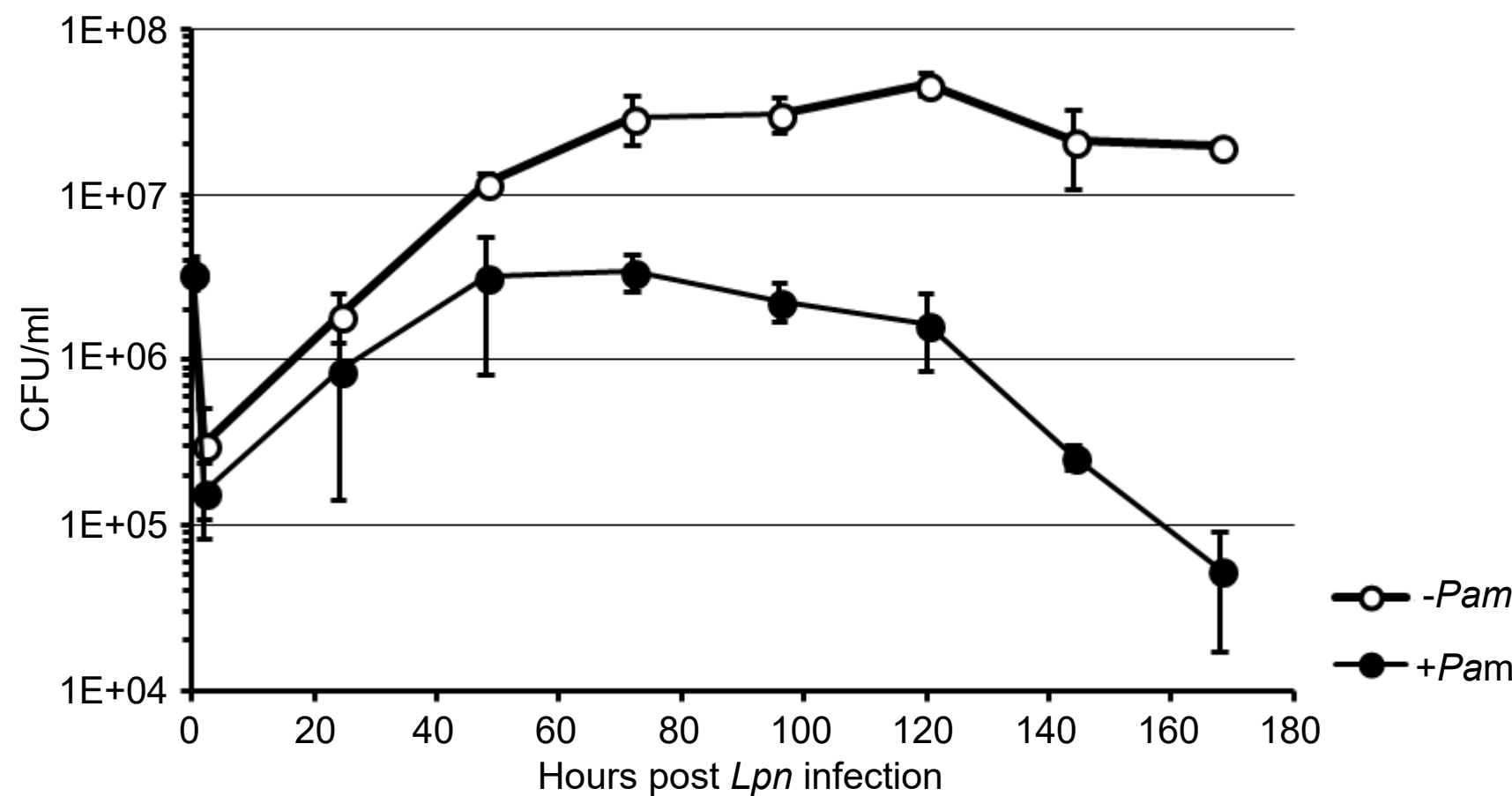

Supplement: FIG S2 [file mBio.00333-19-sf002.pdf]

**A**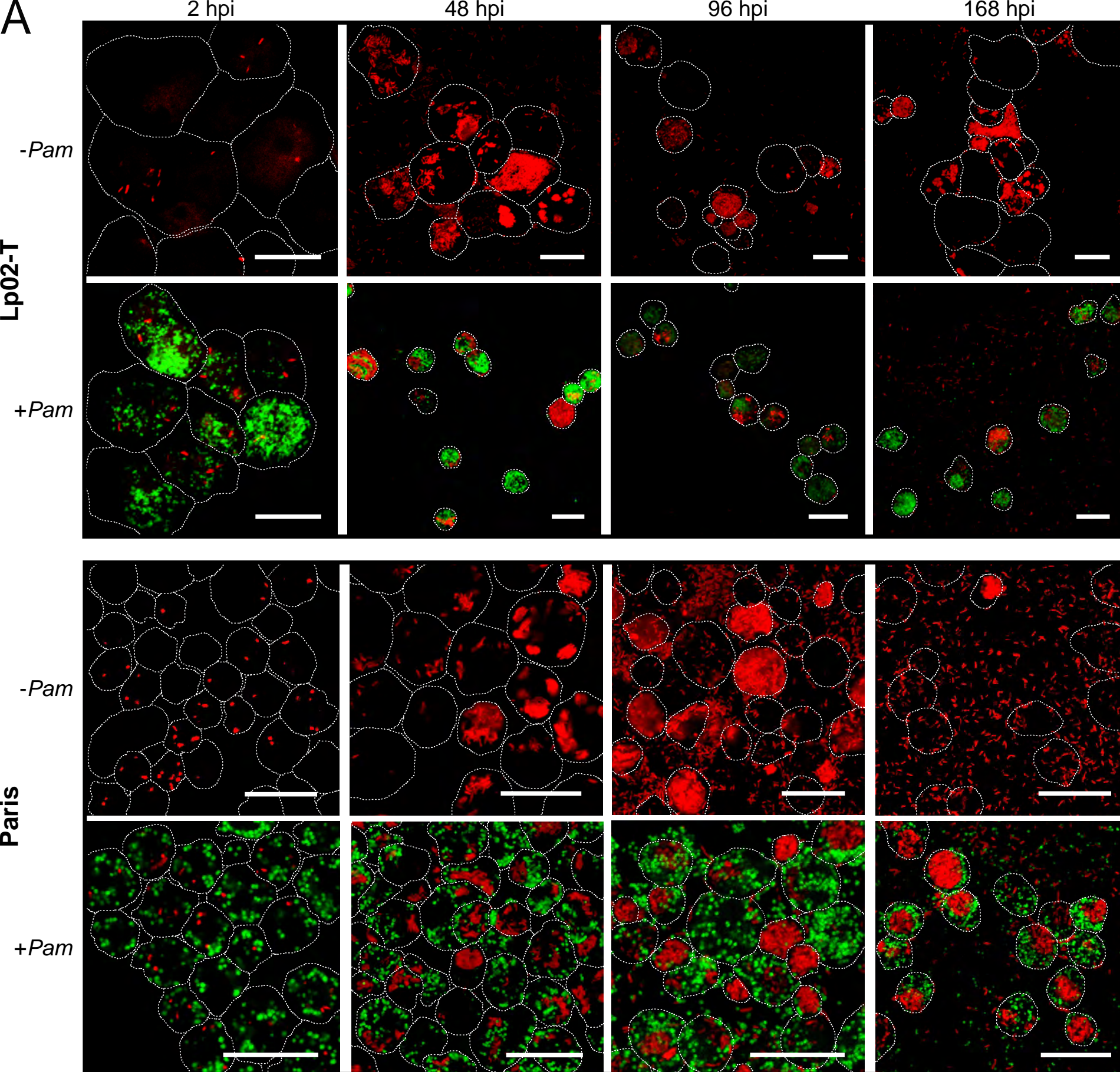**B**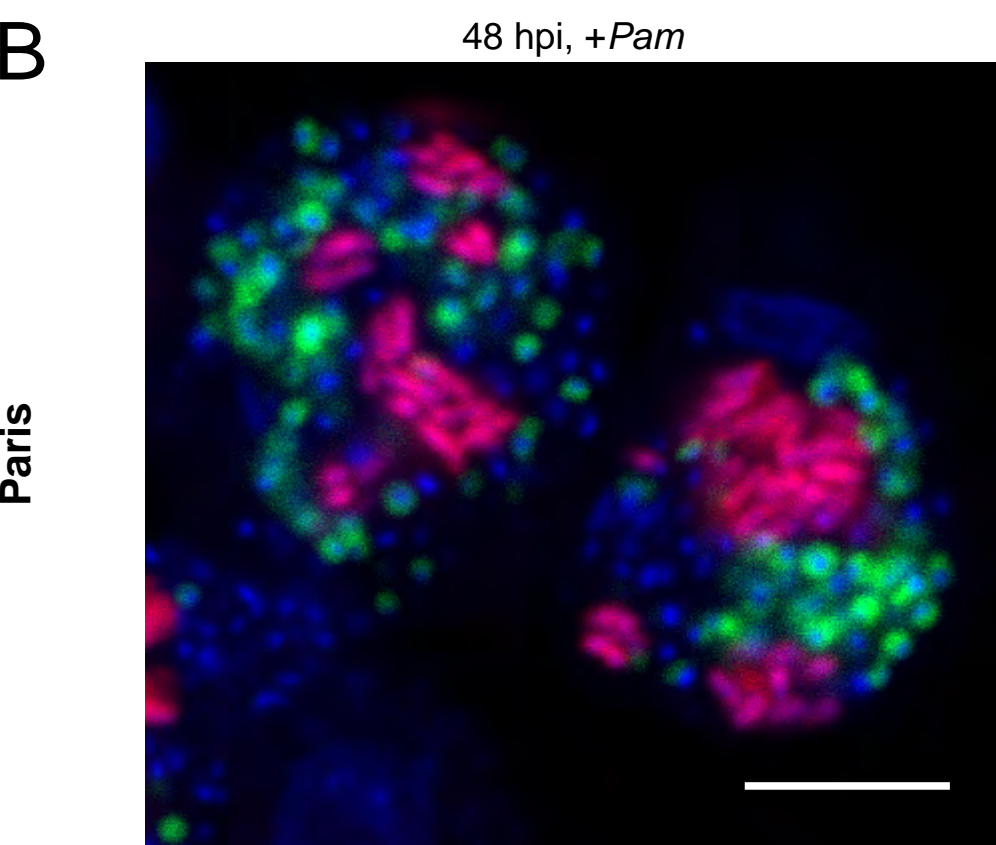

Supplement: FIG S3 [file mBio.00333-19-sf003.pdf]

*A. castellanii* Neff  
+ Paris

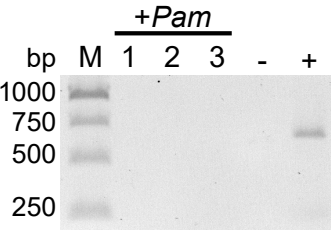

*Acanthamoeba* sp. 2HH  
+ Lp02-T

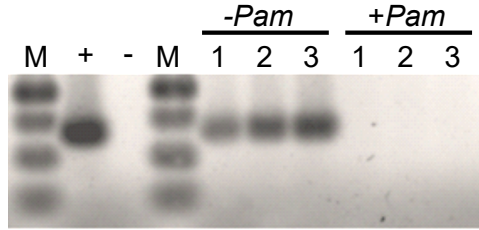

*Acanthamoeba* sp. ML  
+ Lp02-T

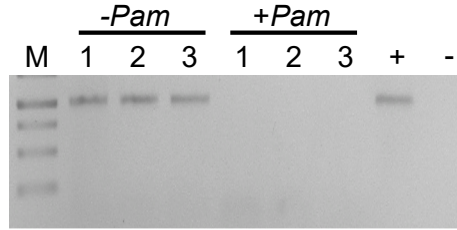

Supplement: FIG S4 [file mBio.00333-19-sf004.pdf]

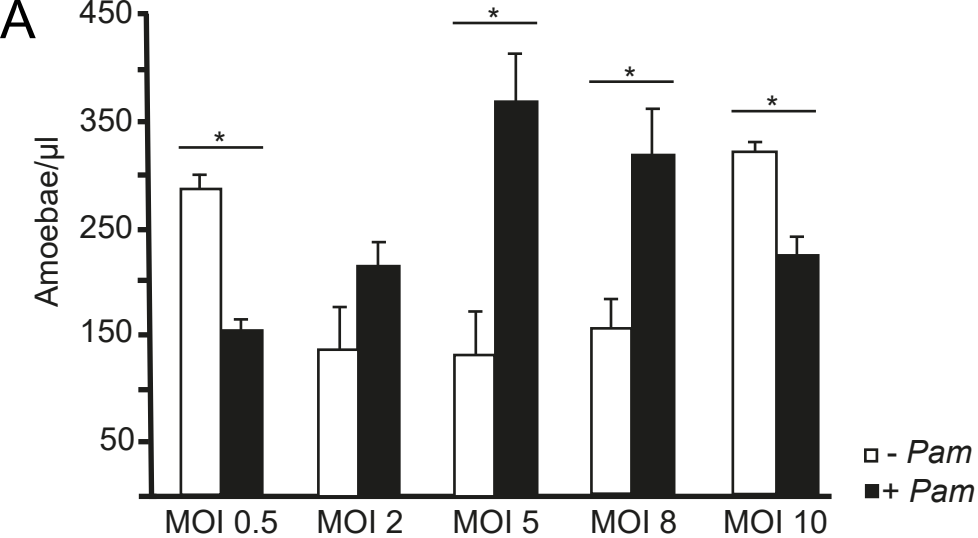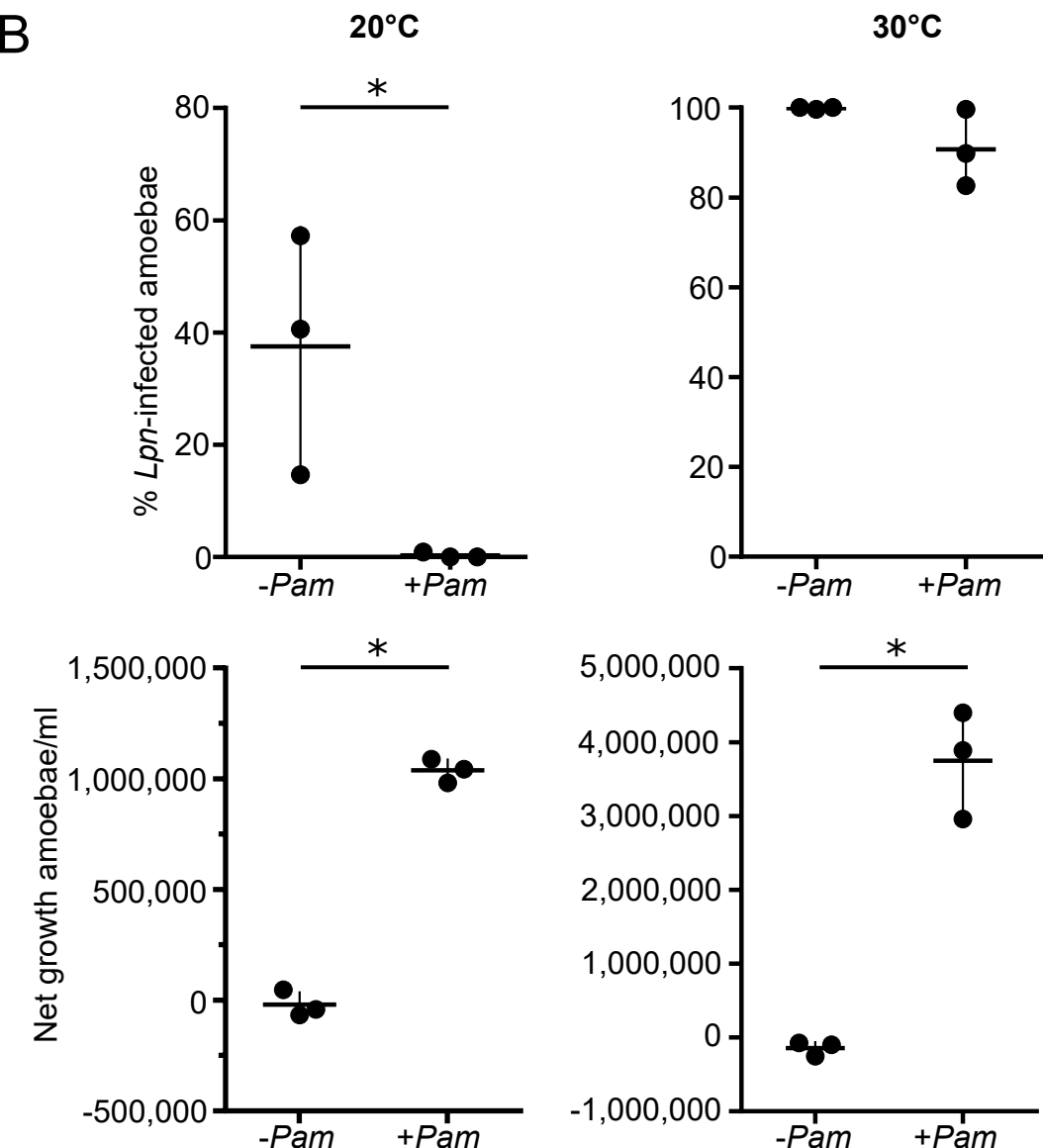

Supplement: FIG S5 [file mBio.00333-19-sf005.pdf]

# A *P. amoebophila*

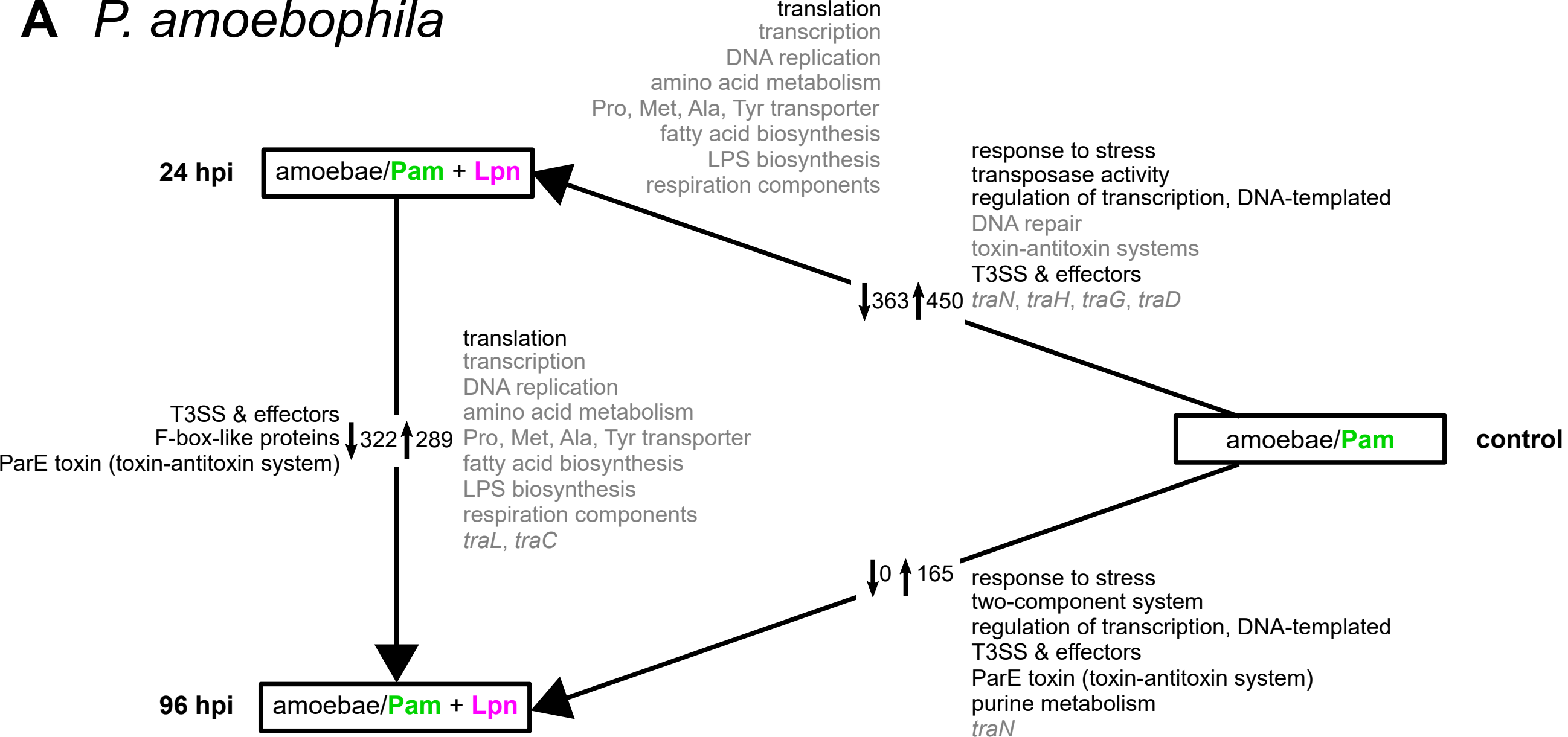

# B *L. pneumophila* Paris

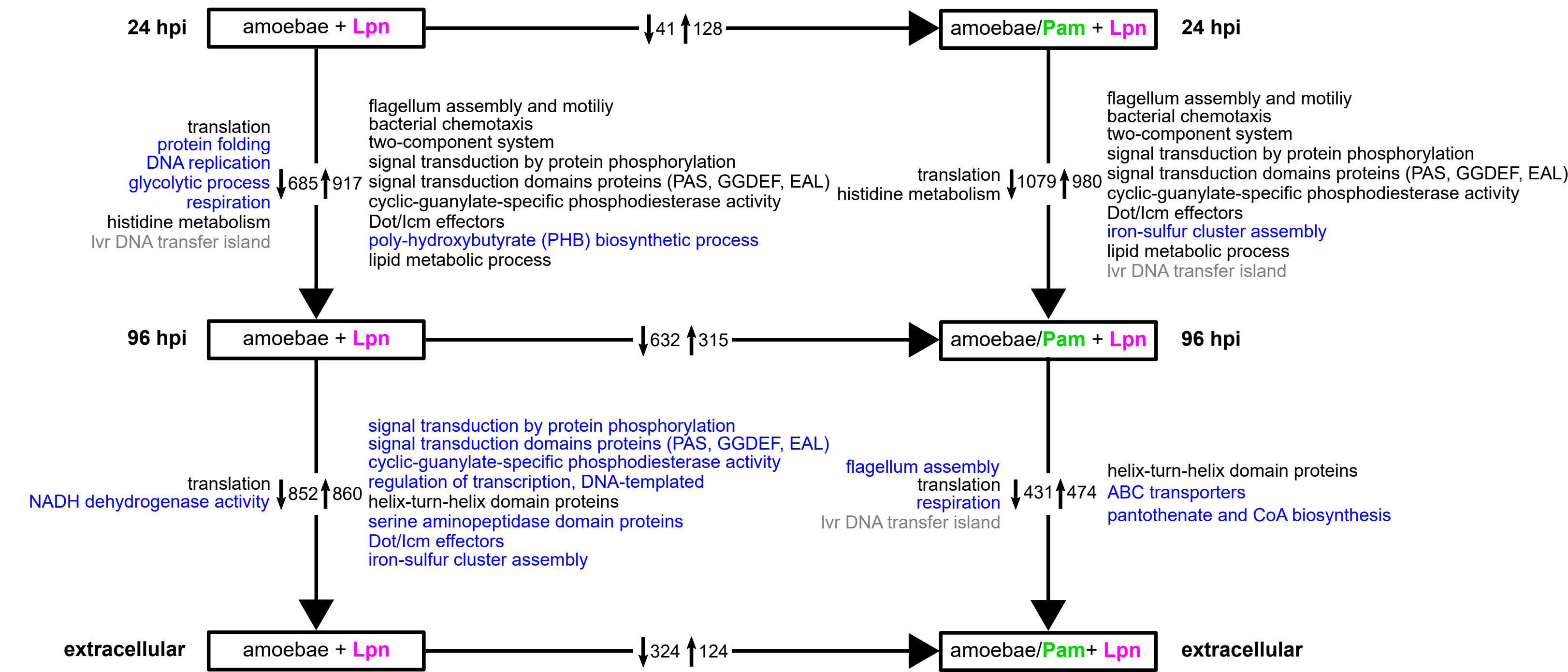

Supplement: FIG S6 [file mBio.00333-19-sf006.pdf]
