# Supplementary material for: Symbiont-Mediated Defense against Legionella pneumophila in Amoebae
Source: mBio. 2019 May 14;10(3):e00333-19. doi: 10.1128/mBio.00333-19 (PMC6520448; doi:10.1128/mBio.00333-19)
Supplement: TEXT S1 [file mBio.00333-19-s0001.docx]

**Text S1. Supplemental Methods and Materials**

**Amoebae growth curves.** Defined numbers of amoebae harvested from continuous cultures of *A. castellanii* Neff with or without the endosymbiont *P. amoebophila* were seeded into PYG-containing multi-well culture plates (Nalge Nunc) at low densities, and amoebae were incubated at 20°C or 30°C. Each day post-seeding, random phase-contrast images per condition and replicate were recorded using a Leica DM IL LED inverted microscope (Leica Microsystems, Wetzlar, Germany) and a Canon EOS 600D camera (Canon Europe Ltd, Uxbridge, UK), and amoeba cells per image were enumerated manually. Numbers of amoebae per surface area and replicate were calculated as the average of ten images. The experiment was conducted in biological triplicates.

**Spread of *P. amoebophila* within endosymbiont-free amoeba cultures.** Uninfected *A. castellanii* Neff and continuous *A. castellanii* Neff*-P. amoebophila* cultures were harvested, amoebae were counted using a Neubauer counting chamber, and a 10:1 mix was seeded in triplicates per time point into multi-well dishes and incubated at 30°C. At each time point post-seeding (1, 21, 45, 69 h post infection, hpi) amoebae were harvested, counted as above and fixed for FISH. Using a probe targeting *P. amoebophila* (Chls-0523, 1) numbers of *P. amoebophila*-infected amoebae relative to uninfected amoebae at each time point were determined using an epifluorescence microscope (Axioplan 2 imaging, Carl Zeiss) and expressed as proportion of infected amoebae.

**PCR**. Clearance from *L. pneumophila* was examined by PCR targeting the *L. pneumophila* *mip* gene. Endosymbiont-free and uninfected amoebae exposed to *L. pneumophila* were harvested 5 wpi, DNA was extracted using the DNeasy Blood & Tissue Kit (Qiagen, Venlo, Netherlands), and about 100 ng of DNA were used in each PCR reaction along with DNA for the positive control (pure culture of *L. pneumophila*) and a negative control (PCR-grade water). One primer pair amplifies the complete *mi*p gene of most *L. pneumophila* strains and yields an amplicon of 981 bp in size (Lp02mipF: 5’-CATTATTAGGGGCAAGTGTAGAAGG, Lp02mipR: 5’-CTGACACCTTTTATGAAGATCATTAGC). The cycler program was as follows: 1x 94°C for 2 min, 35 cycles at 94°C for 45 secs, 57°C for 45 secs and 72°C for 1 min, 1x 72°C for 10 min. The other primer pair used was designed elsewhere for classification of the genus *Legionella* (Legmip_f/Legmip_r; 2). PCR was conducted as suggested by the authors.

**Supplemental References**

1. Poppert S, Essig A, Marre R, Wagner M, Horn M. 2002. Detection and differentiation of chlamydiae by fluorescence in situ hybridization. Appl Environ Microbiol 68:4081–4089.

2. Ratcliff RM, Lanser JA, Manning PA, Heuzenroeder MW. 1998. Sequence-based classification scheme for the genus Legionella targeting the mip gene. J Clin Microbiol 36:1560–1567.
