# Supplementary material for: Symbiont-Mediated Defense against Legionella pneumophila in Amoebae
Source: mBio. 2019 May 14;10(3):e00333-19. doi: 10.1128/mBio.00333-19 (PMC6520448; doi:10.1128/mBio.00333-19)
Supplement: TABLE S1 [file mBio.00333-19-st001.docx]

**Table S1.** Summary of infection experiments

| *Lpn* strain | MOI^1^ | Temp. | Time | Effect on amoebae numbers at end point^2^ | | Effect on *Lpn* at  end point (+*Pam*)^3^ |
| --- | --- | --- | --- | --- | --- | --- |
|  | | | | −*Pam* | +*Pam* |  |
| *Acanthamoeba castellanii* Neff | | | | | | |
| Paris | 0.5 | 20°C | 1 week | − | −− | 🡇 |
| Paris^4^ | 0.5 | 20°C | 5 weeks | − | + | 🡇🡇 |
| Paris | 2 | 20°C | 1 week | −− | − | 🡇 |
| Paris | 5 | 20°C | 1 week | −− | − | 🡇 |
| Paris | 8 | 20°C | 1 week | −− | − | 🡇 |
| Paris | 10 | 20°C | 1 week | ~ | ~ | 🡇 |
| Paris | 20 | 30°C | 1 week | −− | −− | 🡇 |
| Lp02-T | 20 | 20°C | 1 week | ~ | − | 🡇 |
| Lp02-T | 20 | 20°C | 5 weeks | − | − | 🡇 |
| Lp02-T^5^ | 20 | 30°C | 5 days | − | + | 🡇 |
| Lp02-T | 20 | 30°C | 1 week | ~ | ~ | 🡇 |
| Lp02-T | 20 | 30°C | 5 weeks | − | + | 🡇 |
| *Acanthamoeba* sp. ML | | | | | | |
| Paris | 0.5 | 20°C | 1 week | − | −− | 🡇 |
| Paris | 2 | 20°C | 1 week | −− | − | 🡇 |
| Paris | 5 | 20°C | 1 week | −− | − | 🡇 |
| Lp02-T | 20 | 20°C | 5 weeks | + | + | 🡇🡇 |
| Lp02-T | 20 | 20°C | 5 weeks | ~ | + | 🡇🡇 |
| Lp02-T | 20 | 30°C | 5 weeks | ~ | + | 🡇 |
| Lp02-T | 30 | 20°C | 5 days | − | ~ | 🡇 |
| 3626/10 | 20 | 20°C | 5 weeks | n.d. | n.d. | 🡇🡇 |
| 3621 | 20 | 20°C | 5 weeks | n.d. | n.d. | 🡇🡇 |
| *Acanthamoeba* sp. 2HH | | | | | | |
| Lp02-T | 20 | 20°C | 5 weeks | n.d. | n.d. | 🡇🡇 |

^1^ MOIs based on OD_600_ measurements for *Lpn* cultures and numbers of amoebae at the beginning of the infection experiment.

^2^ +, net growth; −, net decline; −−, strong net decline; ~, unchanged. Unless stated otherwise, the effect on amoebae growth was assessed by quantifying amoebae/ml using a hemocytometer. In all cases in which amoebae numbers were not determined, amoeba cells were still present at the end of the experiment.

^3^ 🡇🡇, complete eradication; 🡇, *Lpn* numbers and/or infection load reduced compared to control without the symbiont. Note, that we never observed eradication of *Lpn* in the absence of the symbiont. The color code indicates the method(s) applied: FISH, *Lpn* plate counts, & PCR; FISH & PCR; FISH & *Lpn* plate counts; *Lpn* plate counts; FISH.

^4^ Long-term infection experiments usually involved exchange of PYG medium once a week. However, in this long-term infection experiment amoebae were harvested, collected by centrifugation and resuspended in fresh PYG medium every week (see *Materials and Methods*).

^5^ Amoebae/surface area instead of amoebae/ml were determined (Text S1)

Abbreviations: *Lpn*, *L. pneumophila*, -*Pam*, without endosymbiont; +*Pam*, with endosymbiont; n.d., not determined; Temp., temperature.
