# Supplementary material for: Symbiont-Mediated Defense against Legionella pneumophila in Amoebae
Source: mBio. 2019 May 14;10(3):e00333-19. doi: 10.1128/mBio.00333-19 (PMC6520448; doi:10.1128/mBio.00333-19)
Supplement: TABLE S2 [file mBio.00333-19-st002.docx]

**Table S2.** RNA-seq read and mapping statistics.

|  | Sample | Total number of reads^1^ | Number of reads mapped to genome^2^ | % mapped to genome | % mapped to genes^3^ | Number of reads mapped to rRNA genes | % genes expressed^4^ |
| --- | --- | --- | --- | --- | --- | --- | --- |
| Uninfected control | Neff/Pam-24hpi-1 | 37,787,857 | **Pam**: 13,995,902 | 37.0 | 93.7 | 53,346 | 97.5 |
|  | Neff/Pam-24hpi-2 | 37,674,330 | **Pam**: 17,009,336 | 45.1 | 93.4 | 22,904 | 98.0 |
|  | Neff/Pam-96hpi-1 | 38,384,507 | **Pam**: 12,459,685 | 32.5 | 93.1 | 41,524 | 97.5 |
|  | Neff/Pam-96hpi-2 | 39,135,666 | **Pam**: 13,027,736 | 33.3 | 93.0 | 64,269 | 97.8 |
| *Lpn*-infected, *Pam* absent | Neff+Lpn-24hpi-1 | 35,228,435 | **Lpn**: 6,713,886 | 19.1 | 96.7 | 95 | 92.0 |
|  | Neff+Lpn-24hpi-2 | 38,370,690 | **Lpn**: 6,689,371 | 17.4 | 97.1 | 374 | 91.3 |
|  | Neff+Lpn-24hpi-3 | 39,601,531 | **Lpn**: 10,113,024 | 25.5 | 96.6 | 129 | 93.6 |
|  | Neff+Lpn-96hpi-1 | 26,503,750 | **Lpn**: 8,610,481 | 32.5 | 95.5 | 284 | 93.6 |
|  | Neff+Lpn-96hpi-2 | 20,270,240 | **Lpn**: 4,840,021 | 23.9 | 95.5 | 542 | 91.1 |
|  | Neff+Lpn-extra-1 | 20,253,744 | **Lpn**: 14,381,763 | 71.0 | 94.2 | 834 | 94.9 |
|  | Neff+Lpn-extra-2 | 19,724,879 | **Lpn**: 14,285,462 | 72.4 | 94.3 | 775 | 93.6 |
|  | Neff+Lpn-extra-3 | 20,826,803 | **Lpn**: 18,242,840 | 87.6 | 94.0 | 1184 | 93.0 |
| *Lpn*-infected, *Pam* present | Neff/Pam+Lpn-24hpi-1 | 45,386,490 | **Pam**: 10,721,796  **Lpn**: 5,469,976 | 23.6  12.1 | 91.2  96.5 | 60,876  190 | 96.3  91.6 |
|  | Neff/Pam+Lpn-24hpi-2 | 39,615,355 | **Pam**: 9,609,146  **Lpn**: 5,682,047 | 24.3  14.3 | 92.0  96.8 | 82,475  289 | 95.7  91.1 |
|  | Neff/Pam+Lpn-24hpi-3 | 45,024,525 | **Pam**: 11,328,898  **Lpn**: 10,296,556 | 25.2  22.9 | 90.5  96.0 | 17,563  115 | 96.2  94.4 |
|  | Neff/Pam+Lpn-96hpi-1 | 20,075,610 | **Pam**: 6,524,166  **Lpn**: 4,548,962 | 32.5  22.7 | 96.0  95.0 | 74,374  166 | 93.1  86.4 |
|  | Neff/Pam+Lpn-96hpi-2 | 20,271,941 | **Pam**: 7,095,576  **Lpn**: 3,712,903 | 35.0  18.3 | 95.1  95.7 | 57,058  96 | 95.6  82.3 |
|  | Neff/Pam+Lpn-96hpi-3 | 19,156,018 | **Pam**: 6,906,950  **Lpn**: 4,312,710 | 36.1  22.5 | 95.1  95.5 | 20,788  96 | 95.2  86.3 |
|  | Neff/Pam+Lpn-extra-1 | 20,469,487 | **Lpn**: 7,406,874 | 36.2 | 94.2 | 331 | 93.4 |
|  | Neff/Pam+Lpn-extra-2 | 20,825,650 | **Lpn**: 6,426,927 | 30.9 | 94.6 | 342 | 91.4 |
|  | Neff/Pam+Lpn-extra-3 | 23,134,750 | **Lpn**: 7,082,362 | 30.6 | 94.7 | 306 | 93.7 |

^1^Total number of reads after cleaning pipeline.

^2^Number of reads uniquely mapped to respective genomes.

^3^Percentage of mapped reads that map to genes.

^4^Count threshold: 10 reads per gene.
